# Efficacy and safety of oral semaglutide vs sitagliptin in a predominantly Chinese population with type 2 diabetes uncontrolled with metformin: PIONEER 12, a double-blind, Phase IIIa, randomised trial

**DOI:** 10.1007/s00125-024-06133-4

**Published:** 2024-07-10

**Authors:** Linong Ji, Rikke M. Agesen, Stephen C. Bain, Fangming Fu, Sanaz Gabery, Jianlin Geng, Yiming Li, Yibing Lu, Bifen Luo, Wuyan Pang, Yi Tao, for the PIONEER 12 investigators

**Affiliations:** 1 Peking University People’s Hospital, Beijing, China; 2 Novo Nordisk A/S, Søborg, Denmark; 3 Diabetes Research Unit, Swansea University, Swansea, UK; 4 Jinan Central Hospital Affiliated to Shandong First Medical University, Jinan, China; 5 Harrison International Peace Hospital, Hengshui, China; 6 Huashan Hospital, Fudan University, Shanghai, China; 7 Second Affiliated Hospital of Nanjing Medical University, Nanjing, China; 8 Novo Nordisk (China) Pharmaceuticals Co., Ltd., Beijing, China; 9 Huaihe Hospital of Henan University, Kaifeng, Henan China

**Keywords:** GLP-1 analogue, Glycaemic control, Incretin therapy, Phase III, Semaglutide, Type 2 diabetes

## Abstract

**Aims/hypothesis:**

The aim of this study was to assess the efficacy and safety of oral semaglutide vs sitagliptin in a predominantly Chinese population with type 2 diabetes inadequately controlled with metformin treatment.

**Methods:**

The Peptide Innovation for Early Diabetes Treatment (PIONEER) 12 trial was a randomised, double-dummy, active-controlled, parallel-group, Phase IIIa trial conducted over 26 weeks at 90 sites across the China region (including mainland China, Taiwan and Hong Kong) and five other countries. Adults aged ≥18 years (≥20 years in Taiwan) with a diagnosis of type 2 diabetes, HbA_1c_ between 53 and 91 mmol/mol (inclusive) and treated with a stable daily dose of metformin were eligible for inclusion. Participants were randomised (1:1:1:1) using a web-based randomisation system to either once-daily oral semaglutide (3 mg, 7 mg or 14 mg) or once-daily oral sitagliptin 100 mg. Treatment allocation was masked to both participants and investigators. Randomisation was stratified according to whether participants were from the China region or elsewhere. The primary endpoint was change in HbA_1c_ from baseline to week 26. The confirmatory secondary endpoint was change in body weight (kg) from baseline to week 26. All randomised participants were included in the full analysis set (FAS). All participants exposed to at least one dose of trial product were included in the safety analysis (SAS).

**Results:**

Of 1839 participants screened, 1441 were randomly assigned to oral semaglutide 3 mg (*n*=361), 7 mg (*n*=360), 14 mg (*n*=361) or sitagliptin 100 mg (*n*=359) and included in the FAS. A total of 1438 participants were included in the SAS. In total, 75.2% of participants were from the China region. A total of 1372 (95.2%) participants completed the trial and 130 participants prematurely discontinued treatment (8.3%, 8.6% and 15.0% for oral semaglutide 3 mg, 7 mg and 14 mg, respectively; 4.2% for sitagliptin 100 mg). Significantly greater reductions in HbA_1c_ from baseline to week 26 were reported for all doses of oral semaglutide vs sitagliptin 100 mg. For oral semaglutide 3 mg, 7 mg and 14 mg vs sitagliptin 100 mg, the estimated treatment differences (ETDs [95% CI]) were –2 (–4, –1) mmol/mol, –8 (–9, –6) mmol/mol and –11 (–12, –9) mmol/mol, respectively. The corresponding ETDs (95% CI) in percentage points vs sitagliptin 100 mg were –0.2 (–0.3, –0.1), –0.7 (–0.8, –0.6) and –1.0 (–1.1, –0.8), respectively. Reductions in body weight were significantly greater for all doses of oral semaglutide vs sitagliptin 100 mg (ETD [95% CI] –0.9 [–1.4, –0.4] kg, –2.3 [–2.8, –1.8] kg and –3.3 [–3.8, –2.8] kg for 3 mg, 7 mg and 14 mg, respectively). In the subpopulation of participants from the China region (75.2% of trial participants), reductions in HbA_1c_ and body weight from baseline to week 26 were similar to those seen in the overall population. The most frequent adverse events in the semaglutide treatment arms were gastrointestinal, although these were mostly transient and mild/moderate in severity.

**Conclusions/interpretation:**

Significantly greater reductions in both HbA_1c_ and body weight over 26 weeks were seen with oral semaglutide 3 mg, 7 mg and 14 mg than with sitagliptin 100 mg in a predominantly Chinese population with type 2 diabetes inadequately controlled with metformin treatment. Oral semaglutide was generally well tolerated, with a safety profile consistent with that seen in the global PIONEER trials.

**Trial registration:**

ClinicalTrials.gov NCT04017832.

**Funding:**

This trial was funded by Novo Nordisk A/S, Søborg, Denmark.

**Graphical Abstract:**

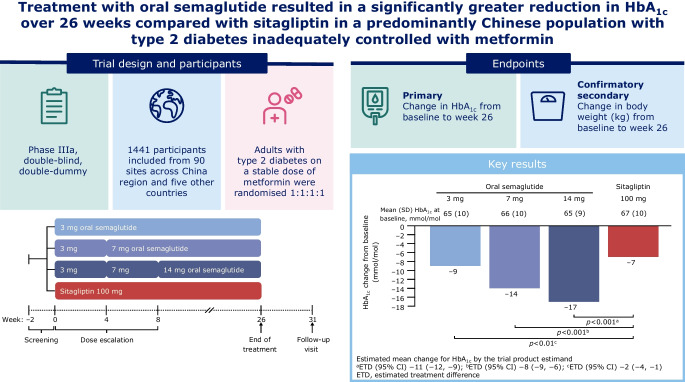

**Supplementary Information:**

The online version contains peer-reviewed but unedited supplementary material available at 10.1007/s00125-024-06133-4.


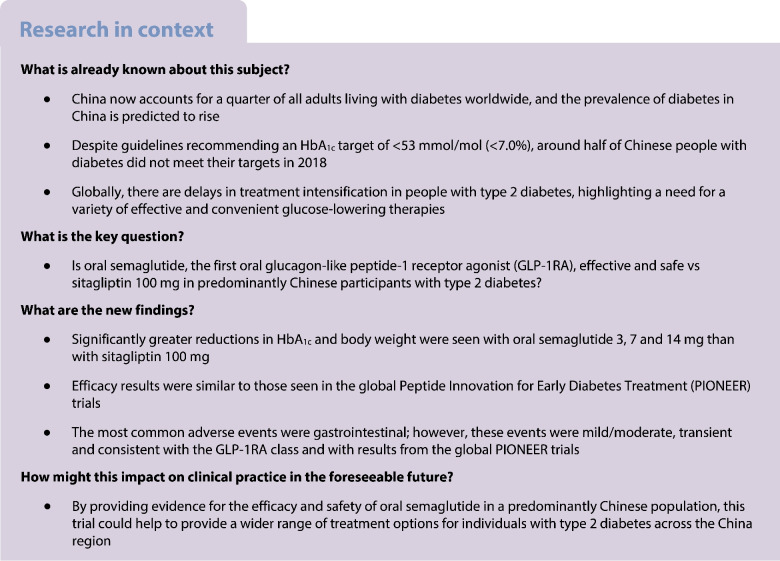


## Introduction

As one of the most common chronic diseases, diabetes is a major health problem both in China and worldwide. As of 2021, a total of 140.9 million people in China had been diagnosed with diabetes, with this number predicted to rise to 174.4 million by 2045 [1]. China now accounts for a quarter of all adults living with diabetes worldwide [1]; hence, there is a need for effective treatment approaches for Chinese populations.

To prevent long-term microvascular and macrovascular complications associated with hyperglycaemia, achieving and maintaining glycaemic control remains the primary goal of type 2 diabetes treatment. Chinese Diabetes Society (CDS), American Diabetes Association (ADA) and ADA/European Association for the Study of Diabetes (EASD) guidelines all recommend an HbA_1c_ target of <53 mmol/mol (<7.0%) [2–4]. However, despite lifestyle modification and the availability of numerous pharmacotherapies, in 2018, only 50.1% of Chinese people with diabetes on glucose-lowering therapies had HbA_1c_ levels within the target range [5].

Overweight and obesity are well-known risk factors for type 2 diabetes. In China, 34.3% and 16.4% of adults had overweight or obesity, respectively, between 2015 and 2019 [6]. CDS guidelines recommend a target of 5–10% body weight loss in people with overweight or obesity [2]. Glucose-lowering therapies that reduce body weight can therefore provide additional clinical benefits in people with type 2 diabetes, with weight loss shown to improve glycaemic control [7]. Glucagon-like peptide-1 receptor agonists (GLP-1RAs) are a drug class capable of reducing both HbA_1c_ and body weight in people with type 2 diabetes [8]. The most recent CDS guidelines recommend the use of GLP-1RAs in combination with metformin, regardless of glycaemic targets, in people with type 2 diabetes with established atherosclerotic CVD or who are at high risk of CVD [2]. It is also recommended that people with type 2 diabetes and overweight or obesity consider treatment with GLP-1RAs or other glucose-lowering drugs with weight loss effects [2].

Globally, there are delays in treatment intensification in people with type 2 diabetes, with one systematic review of therapeutic inertia reporting a median time to treatment intensification ranging from 0.3 to 2.7 years after at least one HbA_1c_ measurement above target [9]. Studies of GLP-1RAs have shown that the mode of administration associated with this drug class (injection) is a barrier to initiation [10], highlighting a need for a variety of effective therapies administered by different routes in this population.

Oral semaglutide, the first GLP-1RA developed for oral administration [11], may be a new option for people experiencing clinical inertia with injectable GLP-1RAs and other currently available therapies [10]. The global Phase IIIa Peptide Innovation for Early Diabetes Treatment (PIONEER) 1–8 trials assessed the efficacy and safety of oral semaglutide in people with type 2 diabetes across a broad range of background medications and comorbidities [12–19]. Oral semaglutide was shown to be effective at improving glycaemic control, with a safety profile consistent with that of the GLP-1RA class [12–19]. Although Asian racial and ethnic groups were included in the PIONEER trials [12–19], and the efficacy and safety of oral semaglutide in a predominantly Japanese population were demonstrated during PIONEER 9 and 10 [20, 21], there is limited evidence available for oral semaglutide from a predominantly Chinese population.

The multiregional PIONEER 12 trial aimed to assess the efficacy and safety of oral semaglutide vs the dipeptidyl peptidase-4 inhibitor (DPP-4i) sitagliptin in a predominantly Chinese population with type 2 diabetes inadequately controlled with metformin.

## Methods

### Trial design

PIONEER 12 (ClinicalTrials.gov NCT04017832) was a 26 week, randomised, double-blind, double-dummy, active-controlled, parallel-group Phase IIIa trial conducted at 90 sites across the China region (including mainland China, Taiwan and Hong Kong) and Brazil, the Czech Republic, Romania, Serbia and South Africa. The trial protocol was approved by the appropriate health authorities according to local guidelines and by an institutional review board/independent ethics committee. The trial was conducted in accordance with the Declaration of Helsinki 2013 and International Council for Harmonisation Good Clinical Practice guidelines. A list of investigators is provided in electronic supplementary material (ESM) Appendix 1. Written informed consent was obtained from all participants prior to any trial-related activities.

### Participants

Participants were aged ≥18 years (≥20 years in Taiwan), had been diagnosed with type 2 diabetes ≥60 days prior to screening, had HbA_1c_ 53–91 mmol/mol inclusive (7.0–10.5% inclusive) and had been on a stable dose of metformin (≥1500 mg or the maximum tolerated dose) for ≥60 days prior to screening. Exclusion criteria included treatment with any other medication for diabetes or obesity in the 60 days prior to screening (except metformin or short-term [14 days] insulin usage), history of pancreatitis, renal impairment and unstable diabetic retinopathy or maculopathy. Full eligibility criteria are provided in ESM Table 1. Data regarding sex, race and ethnicity were self-reported by participants.

### Procedures, randomisation and masking

Following a 2 week screening period, eligible participants were randomised 1:1:1:1 using a web-based randomisation system to once-daily oral semaglutide (3 mg, 7 mg or 14 mg) or once-daily oral sitagliptin (100 mg) for 26 weeks, with the follow-up visit taking place at 31 weeks (ESM Fig. 1). Randomisation was stratified according to whether participants were from the China region or elsewhere. The population of participants from the China region is referred to hereafter as the Chinese subpopulation. The study was double-blinded, meaning that both participants and investigators were masked to the treatment allocation. As oral semaglutide and sitagliptin are not visually identical, a double-dummy trial design was used to mask treatment allocation. This meant that participants allocated to oral semaglutide also received a sitagliptin placebo, and participants allocated to sitagliptin also received an oral semaglutide placebo.

All participants underwent an 8 week escalation regimen to support the double-blinded trial design. Oral semaglutide was initiated at 3 mg and escalated every 4 weeks to the next dose until the randomisation dose was achieved (3 mg, 7 mg or 14 mg). No dose escalation was required for sitagliptin. Background glucose-lowering medication was limited to concomitant metformin only, maintained at the same dose level and frequency as at enrolment, unless rescue medication was needed.

Participants with persistent and unacceptable hyperglycaemia received rescue medication if confirmatory fasting plasma glucose (FPG) tests reported values of >14.4 mmol/l from week 8 to the end of week 13, or >13.3 mmol/l from week 14 to the end of treatment at week 26 (including self-monitored plasma glucose [SMPG]), and no intercurrent cause of the hyperglycaemia could be identified. Rescue medication was prescribed at the investigators’ discretion according to ADA/EASD guidelines [22, 23]. Use of GLP-1RAs, DPP-4is or amylin analogues as rescue medication was not permitted.

### Endpoints and assessments

The primary endpoint was change in HbA_1c_ from baseline to week 26, and the confirmatory secondary endpoint was change in body weight (kg) from baseline to week 26. Supportive secondary endpoints included change from baseline to week 26 in FPG, 7-point SMPG, body weight (%), BMI, waist circumference and fasting lipid profile. Additional supportive secondary endpoints included achievement of HbA_1c_ <53 mmol/mol (<7.0%; ADA target), HbA_1c_ ≤48 mmol/mol (≤6.5%; American Association of Clinical Endocrinologists [AACE] target), an HbA_1c_ reduction ≥10.9 mmol/mol (≥1 percentage point) or body weight loss ≥3%, ≥5 or ≥10% at week 26. Two composite endpoints of HbA_1c_ <53 mmol/mol (<7.0%) without hypoglycaemia (treatment-emergent severe or blood glucose-confirmed symptomatic hypoglycaemia, confirmed by a glucose value <3.1 mmol/l with symptoms consistent with hypoglycaemia) and with no body weight gain, and an HbA_1c_ reduction ≥10.9 mmol/mol (≥1 percentage point) with body weight loss ≥3% were also assessed at week 26. The change from baseline to week 26 in patient-reported outcomes was also evaluated using the 36-item Short Form Health Survey (Acute Version) (SF-36v2).

Safety endpoints included the number of treatment-emergent adverse events (AEs) and the number of hypoglycaemic episodes up to approximately 31 weeks, defined according to the three-tier ADA 2018 classification [23], and the change from baseline to week 26 in laboratory assessments and vital signs.

Information on protocol deviations as a result of the COVID-19 pandemic is provided in ESM Appendix 2.

### Statistical analysis

A sample size of 361 participants per treatment arm was calculated to provide 85% power to jointly confirm the superiority of oral semaglutide (14 mg and 7 mg) vs sitagliptin 100 mg and the non-inferiority of oral semaglutide 3 mg vs sitagliptin 100 mg in reducing HbA_1c_ at week 26 for the trial product estimand. The efficacy endpoint analyses were based on the full analysis set (FAS), which included all randomised participants; analyses of safety endpoints were based on the safety analysis set (SAS), which included all participants exposed to at least one dose of trial product.

Two questions relating to the efficacy objectives were addressed through the definition of two estimands. The trial product (primary) estimand evaluated the treatment effect for all randomised participants under the assumption that all participants continued taking the trial product for the entire planned duration of the trial and did not use rescue medication. The treatment policy (secondary) estimand evaluated the treatment effect for all randomised participants regardless of trial product discontinuation or use of rescue medication. A series of observation periods was defined (ESM Appendix 3).

The primary analysis for the trial product estimand was carried out using a mixed model for repeated measurements using a restricted maximum likelihood, with treatment and region as categorical fixed effects and baseline HbA_1c_ or body weight (depending on the analysis) as covariate. For the trial product estimand, a closed testing procedure was used to control the overall type 1 error at a nominal two-sided 5% level. Overall significance of 0.05 (two-sided) was initially allocated to the HbA_1c_ non-inferiority test of oral semaglutide 14 mg vs sitagliptin 100 mg (ESM Fig. 2). The prespecified margin for assessment of the non-inferiority of semaglutide 3 mg compared with sitagliptin was 0.3%. The statistical testing strategy ensured that non-inferiority was established in terms of HbA_1c_ before testing for superiority and the benefits on body weight at the same dose. Superiority for HbA_1c_ had to be established at higher doses before testing hypotheses at lower doses (ESM Fig. 2). The local significance level was to be reallocated if a hypothesis was confirmed. Further details regarding the statistical analyses performed are provided in ESM Appendix 3 and ESM Fig. 2.

All analyses of the primary and secondary endpoints described above were repeated for the Chinese subpopulation, except for the removal of region as a categorical fixed effect in the model (prespecified).

## Results

### Participants and baseline characteristics

**Fig. 1:**
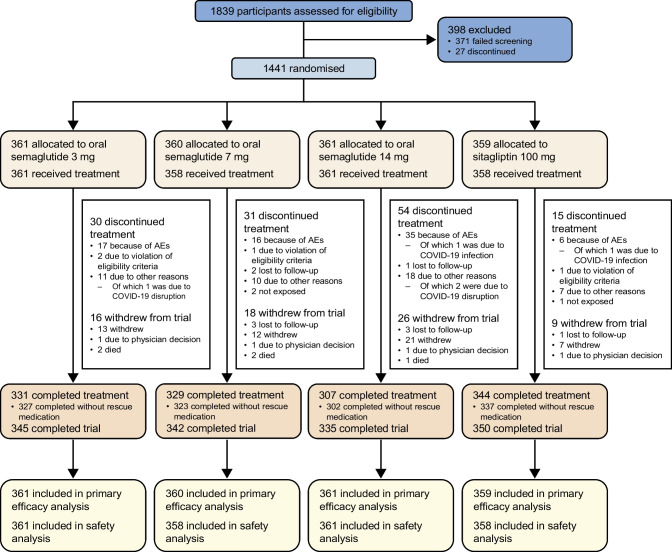
Participant flow diagram

Between July 2019 and October 2021, 1839 individuals were screened, with 1441 randomly assigned to oral semaglutide 3 mg (*n*=361), 7 mg (*n*=360) or 14 mg (*n*=361) or sitagliptin 100 mg (*n*=359). All randomised participants were included in the FAS, and all except for two participants in the oral semaglutide 7 mg arm and one participant in the sitagliptin 100 mg arm were exposed to the trial product and included in the SAS (Fig. 1). The trial was completed by 95.2% (*n*=1372) of participants. The trial product was prematurely discontinued by 8.3% (*n*=30), 8.6% (*n*=31) and 15.0% (*n*=54) of participants in the oral semaglutide 3 mg, 7 mg and 14 mg arms, respectively, and by 4.2% (*n*=15) of participants in the sitagliptin 100 mg arm (Fig. 1). Two participants (0.1%) prematurely discontinued the trial product because of COVID-19 infection, and three participants (0.2%) prematurely discontinued the trial product because of disruption caused by the COVID-19 pandemic. Otherwise, the pandemic and related restrictions did not affect the conduct of the trial and are not considered to have impacted the trial results.

Baseline characteristics were similar across treatment groups (Table 1). Just over half (58.3% [*n*=840]) of all participants were male and 75.2% were from the China region. The mean age of participants was 53 years, mean HbA_1c_ was 66 mmol/mol (8.2%), mean duration of diabetes was 5.6 years, mean FPG was 9.1 mmol/l and mean body weight was 79.5 kg. All participants except for one in the sitagliptin arm were receiving only background metformin at randomisation.

**Table 1:** Participant baseline characteristics (overall population)

Characteristic	Oral semaglutide			Sitagliptin
	3 mg (*N*=361)	7 mg (*N*=360)	14 mg (*N*=361)	100 mg (*N*=359)
Sex, *n* (%)				
Female	152 (42.1)	147 (40.8)	146 (40.4)	156 (43.5)
Male	209 (57.9)	213 (59.2)	215 (59.6)	203 (56.5)
Age, years	53 (11)	53 (11)	54 (10)	53 (11)
Race and ethnicity, *n* (%)				
Asian	272 (75.3)	270 (75.0)	271 (75.1)	271 (75.5)
White	73 (20.2)	65 (18.1)	67 (18.6)	59 (16.4)
Black/African American	8 (2.2)	14 (3.9)	5 (1.4)	11 (3.1)
Other^a^	8 (2.2)	11 (3.1)	18 (5.0)	18 (5.0)
Country/region, *n* (%)				
China region (China, Taiwan and Hong Kong)	272 (75.3)	270 (75.0)	271 (75.1)	271 (75.5)
China	239 (66.2)	246 (68.3)	239 (66.2)	240 (66.9)
Taiwan	18 (5.0)	15 (4.2)	20 (5.5)	19 (5.3)
Hong Kong	15 (4.2)	9 (2.5)	12 (3.3)	12 (3.3)
Brazil	17 (4.7)	18 (5.0)	12 (3.3)	18 (5.0)
Czech Republic	10 (2.8)	12 (3.3)	6 (1.7)	12 (3.3)
Romania	16 (4.4)	16 (4.4)	21 (5.8)	13 (3.6)
Serbia	27 (7.5)	19 (5.3)	24 (6.6)	16 (4.5)
South Africa	19 (5.3)	25 (6.9)	27 (7.5)	29 (8.1)
Duration of diabetes, years	5.8 (5.4)	5.2 (4.9)	5.9 (5.2)	5.6 (4.9)
Body weight, kg	80.8 (19.3)	80.1 (17.7)	79.0 (16.7)	78.3 (17.6)
HbA_1c_				
mmol/mol	65 (10)	66 (10)	65 (9)	67 (10)
%	8.1 (0.9)	8.1 (0.9)	8.1 (0.9)	8.2 (0.9)
FPG, mmol/l	9.2 (2.1)	9.0 (2.2)	9.0 (2.5)	9.1 (2.3)
eGFR, ml/min per 1.73 m^2^	103 (14)	103 (16)	103 (14)	105 (13)
BMI, kg/m^2^	28.9 (6.0)	28.8 (5.6)	28.4 (5.0)	28.2 (5.3)
Waist circumference, cm	98.6 (13.9)	97.8 (13.1)	97.2 (12.2)	97.3 (13.1)
Systolic BP, mmHg^b^	131 (16)	132 (15)^c^	130 (14)	130 (14)^c^
Diastolic BP, mmHg^b^	83 (10)	84 (9)^c^	83 (10)	83 (9)^c^

Data are mean (SD) unless otherwise stated

^a^‘Other’ refers to participants who did not identify as Asian, White or Black/African American

^b^Data were collected from the on-treatment observation period

^c^*N*=358

### Primary endpoint

**Fig. 2:**
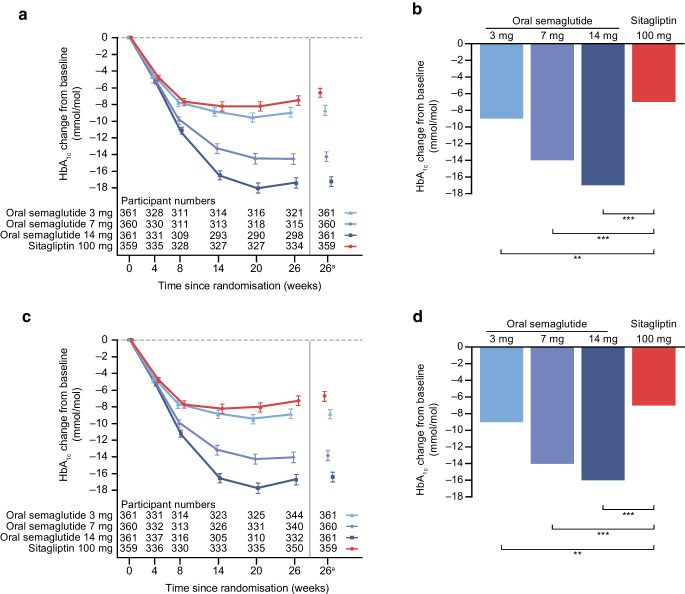
Change in HbA_1c_ from baseline to week 26 (primary endpoint) in the overall trial population. Observed and estimated mean values (±SEM) and estimated mean change from baseline for HbA_1c_ by (**a**, **b**) the trial product estimand and (**c**, **d**) the treatment policy estimand. Data are for the on-treatment without rescue medication (**a**, **b**) or for the in-trial period (**c**, **d**) for the total trial population. At baseline, mean (SD) HbA_1c_ was 65 (10) mmol/mol (8.1% [0.9%]), 66 (10) mmol/mol (8.1% [0.9%]), 65 (9) mmol/mol (8.1% [0.9%]) and 67 (10) mmol/mol (8.2% [0.9%]) for the oral semaglutide 3 mg, 7 mg, 14 mg and sitagliptin 100 mg groups, respectively (FAS). ETDs (95% CI) for oral semaglutide 3, 7 and 14 mg vs sitagliptin 100 mg for the trial product estimand were −2 (−4, −1), −8 (−9, −6) and −11 (−12, −9) mmol/mol, respectively. ETDs (95% CI) for oral semaglutide 3 mg, 7 mg and 14 mg vs sitagliptin 100 mg for the treatment policy estimand were −2 (−4, −1), −7 (−9, −5) and −10 (−11, −8) mmol/mol, respectively. ^a^Estimated means and corresponding error bars are from the primary analysis. **p<0.01; ***p<0.001. ETD, estimated treatment difference

**Fig. 3:**
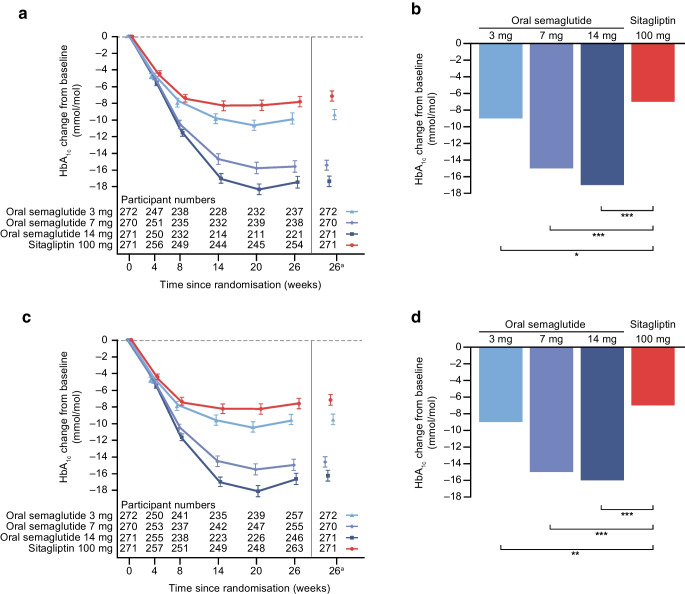
Change in HbA_1c_ from baseline to week 26 (primary endpoint) in the Chinese subpopulation. Observed and estimated mean values (±SEM) and estimated mean change from baseline for HbA_1c_ by (**a**, **b**) the trial product estimand and (**c**, **d**) the treatment policy estimand. Data are for the on-treatment without rescue medication (**a**, **b**) or for the in-trial period (**c**, **d**) for the Chinese subpopulation. At baseline, mean (SD) HbA_1c_ was 65 (10) mmol/mol (8.1% [0.9%]), 66 (10) mmol/mol (8.2% [0.9%]), 65 (9) mmol/mol (8.1% [0.8%]) and 66 (10) mmol/mol (8.2% [0.9%]) for the oral semaglutide 3 mg, 7 mg, 14 mg and sitagliptin 100 mg groups, respectively (Chinese subpopulation FAS). ETDs (95% CI) for oral semaglutide 3 mg, 7 mg and 14 mg vs sitagliptin 100 mg for the trial product estimand were −2 (−4, −1), −8 (−10, −7) and −10 (−12, −8) mmol/mol, respectively. ETDs (95% CI) for oral semaglutide 3 mg, 7 mg and 14 mg vs sitagliptin 100 mg for the treatment policy estimand were −2 (−4, −1), −7 (−9, −6) and −9 (−11, −7) mmol/mol, respectively. ^a^Estimated means and corresponding error bars are from the primary analysis. *p<0.05; **p<0.01; ***p<0.001

For the trial product estimand, oral semaglutide 3 mg, 7 mg and 14 mg were superior to sitagliptin 100 mg in reducing HbA_1c_ from baseline to week 26 (Fig. 2). Estimated mean changes in HbA_1c_ from baseline to week 26 were –9 mmol/mol, –14 mmol/mol and –17 mmol/mol (–0.8 percentage points, –1.3 percentage points and –1.6 percentage points) for oral semaglutide 3 mg, 7 mg and 14 mg, respectively, and –7 mmol/mol (–0.6 percentage points) for sitagliptin 100 mg (Fig. 2a,b). The estimated treatment differences (ETDs [95% CI]) for oral semaglutide 3 mg, 7 mg and 14 mg vs sitagliptin 100 mg were –2 (–4, –1) mmol/mol (*p*<0.01), –8 (–9, –6) mmol/mol and –11 (–12, –9) mmol/mol, respectively (*p*<0.001 for semaglutide 7 mg and 14 mg; Fig. 2b). The corresponding ETDs (95% CI) in percentage points vs sitagliptin 100 mg were –0.2 (–0.3, –0.1), –0.7 (–0.8, –0.6) and –1.0 (–1.1, –0.8), respectively. Similar results were observed for the treatment policy estimand (Fig. 2c,d) and for the Chinese subpopulation for both estimands (Fig. 3).

### Confirmatory secondary endpoint

**Fig. 4:**
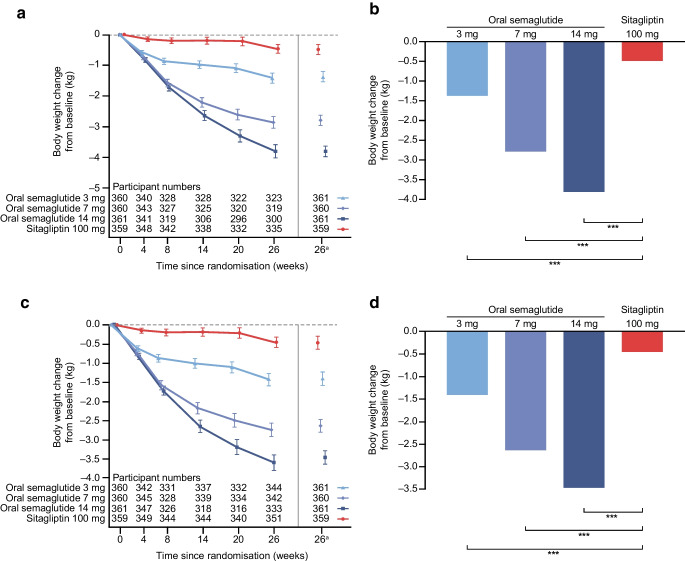
Change in body weight from baseline to week 26 (confirmatory secondary endpoint) in the overall trial population. Observed and estimated mean values (±SEM) and estimated mean change from baseline for body weight by (**a**, **b**) the trial product estimand and (**c**, **d**) the treatment policy estimand. Data are for the on-treatment period without rescue medication (**a**, **b**) or for the in-trial (**c**, **d**) for the total trial population. At baseline, mean (SD) body weight was 80.8 (19.3) kg, 80.1 (17.7) kg, 79.0 (16.7) kg and 78.3 (17.6) kg for the oral semaglutide 3 mg, 7 mg, 14 mg and sitagliptin 100 mg groups, respectively (FAS). ETDs (95% CI) for oral semaglutide 3 mg, 7 mg and 14 mg vs sitagliptin 100 mg for the trial product estimand were −0.9 (−1.4, −0.4), −2.3 (−2.8, −1.8) and −3.3 (−3.8, −2.8) kg, respectively. ETDs (95% CI) for oral semaglutide 3 mg, 7 mg and 14 mg vs sitagliptin 100 mg for the treatment policy estimand were −0.9 (−1.4, −0.5), −2.2 (−2.6, −1.7) and −3.0 (−3.5, −2.5) kg, respectively. ^a^Estimated means and corresponding error bars are from the primary analysis. ***p<0.001

**Fig. 5:**
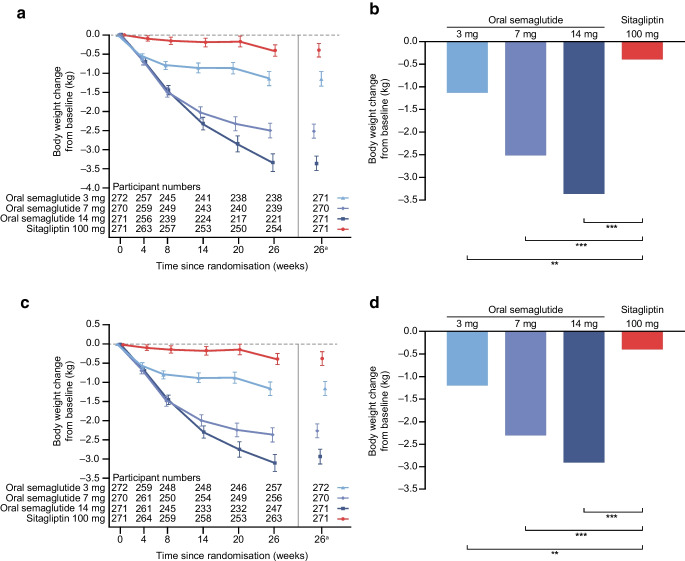
Change in body weight from baseline to week 26 (confirmatory secondary endpoint) in the Chinese subpopulation. Observed and estimated mean values (±SEM) and estimated mean change from baseline for body weight by (**a**, **b**) the trial product estimand and (**c**, **d**) the treatment policy estimand. Data are for the on-treatment period without rescue medication (**a**, **b**) or for the in-trial (**c**, **d**) for the Chinese subpopulation. At baseline, mean (SD) body weight was 75.3 (15.2) kg, 75.0 (13.9) kg, 74.1 (13.7) kg and 73.0 (13.2) kg for the oral semaglutide 3 mg, 7 mg, 14 mg and sitagliptin 100 mg groups, respectively (Chinese subpopulation FAS). ETDs (95% CI) for oral semaglutide 3 mg, 7 mg and 14 mg vs sitagliptin 100 mg for the trial product estimand were −0.8 (−1.3, −0.2), −2.1 (−2.6, −1.6) and −3.0 (−3.5, −2.5) kg, respectively. ETDs (95% CI) for oral semaglutide 3 mg, 7 mg and 14 mg vs sitagliptin 100 mg for the treatment policy estimand were −0.8 (−1.3, −0.3), −1.9 (−2.4, −1.4) and −2.6 (−3.1, −2.1) kg, respectively. ^a^Estimated means and corresponding error bars are from the primary analysis. **p<0.01; ***p<0.001

For the trial product estimand, oral semaglutide 3 mg, 7 mg and 14 mg were superior to sitagliptin 100 mg in reducing body weight from baseline to week 26 (Fig. 4). Estimated mean changes in body weight from baseline to week 26 were –1.4 kg, –2.8 kg and –3.8 kg for oral semaglutide 3 mg, 7 mg and 14 mg, respectively, and –0.5 kg for sitagliptin 100 mg (Fig. 4a,b). ETDs (95% CI) vs sitagliptin 100 mg were –0.9 kg (–1.4 kg, –0.4 kg; *p*<0.001), –2.3 kg (–2.8 kg, –1.8 kg; *p*<0.001) and –3.3 kg (–3.8 kg, –2.8 kg; *p*<0.001), respectively (Fig. 4b). Similar results were observed for the treatment policy estimand (Fig. 4c,d) and for the Chinese subpopulation for both estimands (Fig. 5).

### Supportive secondary endpoints (trial product estimand)

The mean changes in FPG and 7-point SMPG from baseline to 26 weeks were significantly greater with oral semaglutide (all doses) than with sitagliptin 100 mg (Table 2; ESM Fig. 3). The 7-point SMPG postprandial increments were also significantly reduced with oral semaglutide 7 mg and 14 mg vs sitagliptin 100 mg (ESM Table 2). The observed proportions of participants achieving HbA_1c_ <53 mmol/mol (<7.0%; ADA target) and HbA_1c_ ≤48 mmol/mol (≤6.5%; AACE target) were statistically significantly greater with oral semaglutide 7 mg and 14 mg than with sitagliptin 100 mg after 26 weeks of treatment (Table 2).

**Table 2:** Supportive secondary endpoints

Endpoint	Trial product estimand^a^				Treatment policy estimand^b^			
	Oral semaglutide			Sitagliptin	Oral semaglutide			Sitagliptin
	3 mg	7 mg	14 mg	100 mg	3 mg	7 mg	14 mg	100 mg
FPG at week 26, mmol/l								
*N*	360	358	357	353	360	358	357	353
*Nw*	322	317	294	329	342	340	325	344
Estimated mean	8.2	7.3	7.0	8.6	8.1	7.4	7.1	8.6
Change from baseline	–0.9	–1.7	–2.1	–0.5	–1.0	–1.7	–2.0	–0.5
ETD vs sitagliptin (95% CI)	–0.4 (–0.7, –0.1)	–1.3 (–1.6, –1.0)	–1.6 (–1.9, –1.3)	–	–0.5 (–0.7, –0.2)	–1.1 (–1.4, –0.9)	–1.5 (–1.8, –1.2)	–
*p *value	<0.01	<0.001	<0.001	–	<0.01	<0.001	<0.001	–
7-point SMPG at week 26, mmol/l								
*N*	300	311	290	317	357	353	358	351
*Nw*	300	311	290	317	319	333	318	333
Estimated mean	8.9	8.0	7.7	9.2	8.9	8.2	7.8	9.2
Change from baseline	–1.5	–2.4	–2.7	–1.2	–1.5	–2.2	–2.6	–1.2
ETD vs sitagliptin (95% CI)	–0.3 (–0.6, –0.0)	–1.2 (–1.5, –0.9)	–1.5 (–1.8, –1.3)	–	–0.3 (–0.6, –0.0)	–1.0 (–1.3, –0.7)	–1.4 (–1.7, –1.1)	–
*p *value	<0.05	<0.001	<0.001	–	<0.05	<0.001	<0.001	–
HbA_1c_ <53 mmol/mol (<7.0%; ADA target) at week 26								
*N*	361	360	361	359	361	360	361	359
*Nw*	321	315	298	334	344	340	332	350
Proportion of participants, *n* (%)	139 (43.3)	213 (67.6)	226 (75.8)	122 (36.5)	147 (42.7)	219 (64.4)	240 (72.3)	125 (35.7)
OR vs sitagliptin (95% CI)	1.3 (0.9, 1.8)	4.1 (2.9, 5.8)	6.5 (4.5, 9.3)	–	1.2 (0.9, 1.7)	3.3 (2.3, 4.6)	4.8 (3.3, 6.8)	–
*p *value	NS	<0.001	<0.001	–	NS	<0.001	<0.001	–
HbA_1c_ ≤48 mmol/mol (≤6.5%; AACE target) at week 26								
*N*	361	360	361	359	361	360	361	359
*Nw*	321	315	298	334	344	340	332	350
Proportion of participants, *n* (%)	81 (25.2)	161 (51.1)	188 (63.1)	54 (16.2)	85 (24.7)	165 (48.5)	196 (59.0)	54 (15.4)
OR vs sitagliptin (95% CI)	1.7 (1.1, 2.5)	5.8 (3.9, 8.5)	9.9 (6.7, 14.7)	–	1.7 (1.1, 2.5)	5.1 (3.5, 7.5)	7.9 (5.4, 11.6)	–
*p *value	<0.05	<0.001	<0.001	–	<0.05	<0.001	<0.001	–
HbA_1c_ reduction ≥10.9 mmol/mol (≥1 percentage point) at week 26								
*N*	361	360	361	359	361	360	361	359
*Nw*	321	314	298	334	344	339	332	350
Proportion of participants, *n* (%)	141 (43.9)	203 (64.6)	226 (75.8)	129 (38.6)	148 (43.0)	211 (62.2)	239 (72.0)	132 (37.7)
Body weight (%) at week 26								
*N*	360	360	361	359	360	360	361	359
*Nw*	322	319	300	335	343	342	333	351
Change from baseline	–1.7	–3.5	–4.8	–0.5	–1.7	–3.3	–4.3	–0.5
ETD vs sitagliptin (95% CI)	–1.2 (–1.8, –0.6)	–3.1 (–3.6, –2.5)	–4.3 (–4.9, –3.7)	–	–1.2 (–1.8, –0.6)	–2.8 (–3.4, –2.2)	–3.8 (–4.4, –3.2)	–
*p *value	<0.001	<0.001	<0.001	–	<0.001	<0.001	<0.001	–
Body weight loss ≥3% at week 26								
*N*	361	360	361	359	361	360	361	359
*Nw*	321	314	298	334	344	339	332	350
Proportion of participants, *n* (%)	99 (30.8)	166 (52.9)	185 (62.1)	65 (19.5)	109 (31.7)	175 (51.6)	199 (59.9)	68 (19.4)
Body weight loss ≥5% at week 26								
*N*	360	360	361	359	360	360	361	359
*Nw*	322	319	300	335	343	342	333	351
Proportion of participants, *n* (%)	51 (15.8)	106 (33.2)	137 (45.7)	27 (8.1)	55 (16.0)	107 (31.3)	149 (44.7)	28 (8.0)
OR vs sitagliptin (95% CI)	2.3 (1.4, 3.7)	6.0 (3.8, 9.5)	10.0 (6.4, 15.6)	–	2.3 (1.4, 3.7)	5.1 (3.3, 8.0)	8.9 (5.7, 13.8)	–
*p *value	<0.01	<0.001	<0.001	–	<0.001	<0.001	<0.001	–
Body weight loss ≥10% at week 26								
*N*	360	360	361	359	360	360	361	359
*Nw*	322	319	300	335	343	342	333	351
Proportion of participants, *n* (%)	5 (1.6)	23 (7.2)	40 (13.3)	2 (0.6)	5 (1.5)	24 (7.0)	42 (12.6)	2 (0.6)
OR vs sitagliptin (95% CI)	2.9 (0.6, 14.8)	14.0 (3.3, 59.3)	28.2 (6.8, 117.0)	–	2.6 (0.5, 13.3)	11.7 (2.8, 49.2)	22.4 (5.5, 91.7)	–
*p *value	NS	<0.001	<0.001	–	NS	<0.001	<0.001	–
HbA_1c_ <53 mmol/mol (<7.0%) without hypoglycaemia^c^ and no body weight gain at week 26								
*N*	360	360	361	359	360	360	361	359
*Nw*	320	314	298	334	343	339	332	350
Proportion of participants, *n* (%)^d^	109 (34.0)	190 (60.5)	209 (70.1)	76 (22.8)	114 (33.1)	195 (57.5)	222 (66.9)	79 (22.6)
OR vs sitagliptin (95% CI)	1.7 (1.2, 2.5)	5.6 (3.9, 8.0)	8.7 (6.0, 12.6)	–	1.6 (1.1, 2.3)	4.7 (3.3, 6.6)	6.8 (4.7, 9.6)	–
*p *value	<0.01	<0.001	<0.001	–	<0.01	<0.001	<0.001	–
HbA_1c_ reduction ≥10.9 mmol/mol (≥1 percentage point) and body weight loss ≥3% at week 26								
*N*	360	360	361	359	360	360	361	359
*Nw*	320	314	298	334	343	339	332	350
Proportion of participants, *n* (%)^d^	61 (19.0)	123 (39.2)	154 (51.7)	31 (9.3)	64 (18.6)	128 (37.8)	159 (47.9)	33 (9.4)
OR vs sitagliptin (95% CI)	2.4 (1.5, 3.7)	6.6 (4.3, 10.1)	10.6 (6.9, 16.2)	–	2.2 (1.4, 3.5)	5.7 (3.7, 8.6)	8.2 (5.4, 12.4)	–
*p *value	<0.001	<0.001	<0.001	–	<0.001	<0.001	<0.001	–
Total cholesterol (mmol/l) at week 26								
*N*	329	331	310	343	357	358	358	356
*Nw*	316	316	296	330	336	340	328	346
Estimated mean	4.53	4.35	4.32	4.46	4.53	4.35	4.32	4.44
Estimated ratio to baseline	1.00	0.96	0.96	0.99	1.00	0.97	0.96	0.99
ETR vs sitagliptin (95% CI)	1.01 (0.99, 1.04)	0.97 (0.95, 1.00)	0.97 (0.94, 0.99)	–	1.02 (0.99, 1.05)	0.98 (0.96, 1.00)	0.97 (0.95, 1.00)	–
*p *value	NS	<0.05	<0.05	–	NS	NS	<0.05	–
LDL-cholesterol^e^ (mmol/l) at week 26								
*N*	328	330	309	341	356	357	357	354
*Nw*	315	314	295	327	335	339	327	343
Estimated mean	2	2	2	2	2	2	2	2
Estimated ratio to baseline	1.02	0.98	0.98	1.01	1.02	0.98	0.97	1.01
ETR vs sitagliptin (95% CI)	1.00 (0.97, 1.04)	0.97 (0.93, 1.01)	0.96 (0.92, 1.00)	–	1.01 (0.97, 1.05)	0.97 (0.94, 1.01)	0.97 (0.93, 1.01)	–
*p *value	NS	NS	NS	–	NS	NS	NS	–
VLDL-cholesterol^e^ (mmol/l) at week 26								
*N*	328	330	309	341	356	357	357	354
*Nw*	315	315	295	327	335	339	327	343
Estimated mean	0.77	0.69	0.68	0.71	0.76	0.69	0.68	0.71
Estimated ratio to baseline	0.97	0.87	0.87	0.90	0.97	0.88	0.86	0.90
ETR vs sitagliptin (95% CI)	1.07 (1.01, 1.13)	0.96 (0.91, 1.01)	0.96 (0.90, 1.01)	–	1.08 (1.02, 1.14)	0.98 (0.93, 1.04)	0.96 (0.91, 1.02)	–
*p *value	<0.05	NS	NS	–	<0.01	NS	NS	–
Triglycerides (mmol/mol) at week 26								
*N*	328	330	309	341	356	357	357	354
*Nw*	315	315	295	327	335	339	327	343
Estimated mean	1.73	1.53	1.54	1.61	1.72	1.55	1.52	1.59
Estimated ratio to baseline	0.97	0.86	0.86	0.90	0.97	0.87	0.85	0.90
ETR vs sitagliptin (95% CI)	1.07 (1.01, 1.14)	0.95 (0.90, 1.01)	0.96 (0.90, 1.02)	–	1.08 (1.02, 1.15)	0.97 (0.92, 1.03)	0.95 (0.90, 1.01)	–
*p *value	<0.05	NS	NS	–	<0.05	NS	NS	–

^a^The trial product estimand evaluated the treatment difference for all randomised participants assuming that all participants continued taking the trial product for the entire trial duration and did not use rescue medication. Data are from the on-treatment period without rescue medication (from when participants were considered treated with trial product until 3 days after the final dose of trial product or until initiation of rescue medication), and were estimated using a mixed model for repeated measurements and restricted maximum likelihood

^b^The treatment policy estimand evaluates the treatment difference for all randomised participants regardless of trial product discontinuation or use of rescue medication. Data are from the in-trial observation period (from when participants were randomised until the follow-up visit, withdrawal or death) and were estimated using a pattern mixture model using multiple imputation to handle missing data

^c^Hypoglycaemia was defined according to the severe ADA classification [23] or was confirmed by a plasma glucose level <3.1 mmol/l with symptoms consistent with hypoglycaemia

^d^Proportion data for the oral semaglutide 3 mg arm were calculated using the number of participants with non-missing data (*N*=321 for the trial product estimand and *N*=344 for the treatment policy estimand)

^e^Twenty-three data points for LDL-cholesterol and one data point for VLDL-cholesterol were included in the statistical analysis despite exceeding the time period for sample stability. This was noted after the database lock

*ETR* estimated treatment ratio, *N* number of participants contributing to the analysis, *NS* not significant, *Nw* number of participants with an observation at the visit

Significantly greater reductions in body weight (%) at 26 weeks were observed with all doses of oral semaglutide vs sitagliptin (*p*<0.001; Table 2). The observed proportions of participants achieving ≥3%, ≥5% or ≥10% body weight loss at 26 weeks were greater with all oral semaglutide doses than with sitagliptin 100 mg (Table 2). The ORs of achieving ≥5% or ≥10% body weight loss were statistically significant in favour of oral semaglutide 7 mg (*p*<0.001 for both) and 14 mg (*p*<0.001 for both) vs sitagliptin 100 mg (Table 2). For oral semaglutide 3 mg, achievement of ≥5% body weight loss was significant (*p*<0.01) but achievement of ≥10% body weight loss was not significant (Table 2). Significantly greater reductions in BMI and waist circumference were seen with oral semaglutide (all doses) than with sitagliptin 100 mg (*p*<0.05 for all; ESM Table 2).

The ORs of achieving both composite endpoints (i.e. achieving HbA_1c_ <53 mmol/mol [<7.0%] without hypoglycaemia or weight gain and achieving a ≥10.9 mmol/mol [≥1 percentage point] HbA_1c_ reduction and ≥3% weight loss) were statistically significant in favour of oral semaglutide (all doses) vs sitagliptin 100 mg (Table 2).

Estimated treatment ratios to baseline were generally similar between treatment groups across the lipid profile, except for total cholesterol, which was significantly lower in participants treated with oral semaglutide 7 mg and 14 mg than in participants treated with sitagliptin (estimated treatment ratio [trial product estimand] 0.97 [95% CI 0.95, 1.00; *p*<0.05] and 0.97 [0.94, 0.99; *p*<0.05], respectively; Table 2).

Overall, patient-reported outcomes (assessed by the SF-36v2) improved, but there were no statistically significant differences between oral semaglutide and sitagliptin (ESM Table 2). The proportions of participants on additional concomitant and rescue glucose-lowering medication were low and similar across all treatment groups; the time from first dose to rescue medication use was not significantly different between any of the oral semaglutide doses and sitagliptin 100 mg (ESM Table 3).

The results for the treatment policy estimand are also presented in Table 2 and ESM Table 2 and are broadly similar to the results for the trial product estimand.

### Adverse events and tolerability

The overall proportion of participants experiencing AEs while on treatment was slightly higher for oral semaglutide 7 mg and 14 mg than for oral semaglutide 3 mg and sitagliptin 100 mg, with most AEs being mild/moderate in severity (Table 3). Across all semaglutide treatment arms, the most frequent AEs were gastrointestinal disorders (most commonly nausea, diarrhoea and vomiting), the majority of which were mild/moderate in severity. Gastrointestinal disorders were reported by a greater proportion of participants on all doses of oral semaglutide than participants on sitagliptin 100 mg, and increased in frequency as the oral semaglutide dose increased. For sitagliptin 100 mg, the most common AEs were upper respiratory tract infections, diabetic retinopathy and diarrhoea (Table 3). In the Chinese subpopulation, the proportion of participants experiencing AEs was slightly higher across all treatment arms compared with the overall population; however, a higher proportion of total AEs was classed as mild in severity compared with the overall population.

**Table 3:** Adverse events

AE	Oral semaglutide						Sitagliptin	
	3 mg		7 mg		14 mg		100 mg	
	Overall population (*N*=361)	Chinese subpopulation (*N*=272)	Overall population (*N*=358)	Chinese subpopulation (*N*=268)	Overall population (*N*=361)	Chinese subpopulation (*N*=271)	Overall population (*N*=358)	Chinese subpopulation (*N*=270)
Any AE	234 (64.8)	195 (71.7)	257 (71.8)	203 (75.7)	263 (72.9)	209 (77.1)	237 (66.2)	188 (69.6)
AEs by severity								
Mild	221 (61.2)	188 (69.1)	240 (67.0)	197 (73.5)	238 (65.9)	200 (73.8)	219 (61.2)	180 (66.7)
Moderate	44 (12.2)	32 (11.8)	57 (15.9)	36 (13.4)	73 (20.2)	45 (16.6)	56 (15.6)	30 (11.1)
Severe	8 (2.2)	5 (1.8)	12 (3.4)	7 (2.6)	8 (2.2)	6 (2.2)	5 (1.4)	4 (1.5)
SAEs	16 (4.4)	12 (4.4)	11 (3.1)	6 (2.2)	11 (3.0)	9 (3.3)	15 (4.2)	12 (4.4)
AEs leading to trial product discontinuation	17 (4.7)	16 (5.9)	16 (4.5)	15 (5.6)	35 (9.7)	30 (11.1)	6 (1.7)	6 (2.2)
Hypoglycaemic events^a^								
Level 1	9 (2.5)	5 (1.8)	12 (3.4)	7 (2.6)	22 (6.1)	12 (4.4)	8 (2.2)	1 (0.4)
Level 2	5 (1.4)	0	2 (0.6)	0	2 (0.6)	1 (0.4)	0	0
Level 3	0	0	0	0	0	0	0	0
EAC-confirmed deaths	2 (0.6)	1 (0.4)	2 (0.6)	0	1 (0.3)	0	0	0
Most frequent AEs occurring in ≥5% of participants in any treatment group (preferred term)								
Nausea	36 (10.0)	26 (9.6)	39 (10.9)	30 (11.2)	44 (12.2)	32 (11.8)	14 (3.9)	7 (2.6)
Diarrhoea	30 (8.3)	26 (9.6)	32 (8.9)	30 (11.2)	48 (13.3)	40 (14.8)	16 (4.5)	16 (5.9)
Vomiting	16 (4.4)	13 (4.8)	18 (5.0)	15 (5.6)	29 (8.0)	22 (8.1)	1 (0.3)	1 (0.4)
Abdominal distension	7 (1.9)	7 (2.6)	16 (4.5)	16 (6.0)	17 (4.7)	14 (5.2)	4 (1.1)	4 (1.5)
Decreased appetite	22 (6.1)	21 (7.7)	30 (8.4)	27 (10.1)	34 (9.4)	33 (12.2)	5 (1.4)	4 (1.5)
Upper respiratory tract infection	26 (7.2)	26 (9.6)	26 (7.3)	25 (9.3)	31 (8.6)	28 (10.3)	28 (7.8)	27 (10.0)
Increased lipase levels	15 (4.2)	14 (5.1)	17 (4.7)	15 (5.6)	21 (5.8)	19 (7.0)	12 (3.4)	11 (4.1)
Diabetic retinopathy	13 (3.6)	13 (4.8)	11 (3.1)	9 (3.4)	12 (3.3)	12 (4.4)	18 (5.0)	15 (5.6)
Dizziness	12 (3.3)	11 (4.0)	16 (4.5)	14 (5.2)	13 (3.6)	12 (4.4)	5 (1.4)	5 (1.9)

Data are *n* (%), where *n* is the number of participants with an event and % is the proportion of participants with an event

AEs are shown for the SAS (which included all participants exposed to at least one dose of trial product) and are those that occurred during the on-treatment period (the time period during which participants were considered to have been treated with the trial product)

^a^Hypoglycaemia was defined according to the ADA 2018 classification, in which level 1 is defined as an alert value with plasma glucose levels of ≤3.9 mmol/l, level 2 is defined as clinically significant with plasma glucose levels of <3.0 mmol/l and level 3 is defined as severe (no specific glucose threshold) and requires assistance from another person for recovery [23]

EAC, event adjudication committee; SAE, serious adverse event

The proportion of participants with serious adverse events (SAEs) was low in all treatment groups but was highest in the oral semaglutide 3 mg and sitagliptin 100 mg arms; this was also the case for the Chinese subpopulation (Table 3). There were five deaths among exposed participants (of which one was in the Chinese subpopulation): two in the oral semaglutide 3 mg arm from infection (one as a result of acute exacerbation of interstitial lung disease and one from COVID-19 pneumonia), two in the oral semaglutide 7 mg arm from infection and other causes (COVID-19 pneumonia and pulmonary embolism) and one in the oral semaglutide 14 mg arm (undetermined cause). All deaths were judged by the investigators as unlikely to be related to the trial product.

The proportion of participants who prematurely discontinued the trial product because of AEs in both the overall population and the Chinese subpopulation was higher in the oral semaglutide 14 mg arm (9.7% and 11.1%, respectively) than in the oral semaglutide 3 mg (4.7% and 5.9%), oral semaglutide 7 mg (4.5% and 5.6%) and sitagliptin 100 mg (1.7% and 2.2%) arms, with gastrointestinal AEs being the most common reason for discontinuation across all treatment groups. The frequency of hypoglycaemic episodes was low across groups, with 1.4%, 0.6%, 0.6% and 0% of participants experiencing a level 2 hypoglycaemic event in the oral semaglutide 3 mg, 7 mg and 14 mg and sitagliptin 100 mg arms, respectively. Only one participant in the Chinese subpopulation, who was in the oral semaglutide 14 mg arm, experienced a level 2 hypoglycaemic event. No level 3 severe hypoglycaemic events were reported (Table 3).

Eye complications (including diabetic retinopathy) were mild/moderate in severity for both the overall and the Chinese subpopulation. In the overall population they were reported by 4.7% (17/361), 3.6% (13/358), 3.3% (12/361) and 5.6% (20/358) of participants in the oral semaglutide 3 mg, 7 mg, 14 mg and sitagliptin 100 mg arms, respectively. Corresponding values in the Chinese subpopulation were 5.9% (16/272), 3.7% (10/268), 4.4% (12/271) and 7.0% (19/270) of participants, respectively.

The frequencies of event adjudication committee-confirmed events, including acute kidney injury, cardiovascular events and malignant neoplasms, were low and similar across all treatment arms in the overall population (ESM Table 4). There were no event adjudication committee-confirmed cases of acute pancreatitis or medullary thyroid cancer. Treatment with oral semaglutide resulted in mean increases in amylase and lipase levels during the initial 14 weeks of the trial, after which no further increases were observed. Increases in amylase and lipase were statistically significant for oral semaglutide 14 mg vs sitagliptin 100 mg (treatment ratio [95% CI] 1.05 [1.01, 1.09; *p*<0.01] for amylase; 1.11 [1.03, 1.18; *p*<0.01] for lipase). Other safety variables, including laboratory assessments and vital signs, are reported in ESM Table 5.

## Discussion

The results of PIONEER 12 demonstrate that once-daily oral semaglutide was superior to sitagliptin 100 mg in improving glycaemic control and lowering body weight in combination with metformin in a predominantly Chinese population with type 2 diabetes inadequately controlled with metformin. Significantly greater reductions in HbA_1c_ and body weight from baseline to week 26 were observed for all doses of oral semaglutide than for sitagliptin 100 mg using the trial product estimand. Similar reductions were observed in the Chinese subpopulation, which represented 75.2% of the overall population. For the treatment policy estimand, the observed reductions in HbA_1c_ and body weight were dose dependent, a finding that is consistent with the results using the treatment policy estimand in the global PIONEER programme [12–19]. Reductions in HbA_1c_ and body weight from baseline to week 26 in the overall PIONEER 12 population were greater than those observed at week 26 in the PIONEER 3 trial, which assessed oral semaglutide 3 mg, 7 mg and 14 mg vs sitagliptin 100 mg in a global population across 78 weeks [18].

Participants treated with all doses of oral semaglutide experienced greater reductions in FPG and 7-point SMPG than those treated with sitagliptin 100 mg. These greater reductions with oral semaglutide compared with sitagliptin are consistent with the results seen in the global PIONEER 3 population. Furthermore, the reductions in FPG and 7-point SMPG observed with all doses of oral semaglutide at week 26 in PIONEER 12 were greater than those observed with the same doses of oral semaglutide at week 26 during PIONEER 3 [18]. Participants treated with oral semaglutide were more likely to achieve HbA_1c_ <53 mmol/mol (<7.0%), HbA_1c_ ≤48 mmol/mol (≤6.5%), body weight loss of ≥5% and ≥10% and the composite endpoints than those treated with sitagliptin. Reductions in total cholesterol were significantly greater in participants treated with oral semaglutide 7 mg and 14 mg than in those treated with sitagliptin 100 mg (trial product estimand), which is also consistent with the PIONEER 3 trial results [18]. Oral semaglutide was generally well tolerated across the trial treatment period, although a greater proportion of participants experienced AEs leading to premature trial discontinuation with oral semaglutide than with sitagliptin. This was largely due to the greater incidence of gastrointestinal AEs with oral semaglutide; these were the most frequently reported AEs across all treatment arms but they were mostly transient and mild/moderate in severity, consistent with the safety profile of the GLP-1RA drug class [8, 18, 24]. A higher rate of gastrointestinal AEs was observed in PIONEER 12 than in PIONEER 3 [18]. This could potentially be explained by the greater levels of semaglutide exposure as a result of the lower mean baseline body weight in PIONEER 12 than in PIONEER 3 (79.5 kg vs 91.2 kg) [18, 25]. Additionally, PIONEER 12 required administration of sitagliptin or sitagliptin placebo 30 min after administration of oral semaglutide or oral semaglutide placebo, whereas in PIONEER 3 these were administered simultaneously [18]; it is not unreasonable to consider that co-administration of placebo with oral semaglutide could reduce exposure and may potentially explain the increased efficacy in this trial compared with PIONEER 3. Indeed, lower semaglutide exposure was observed in PIONEER 3 than in the other global PIONEER trials [26]; however, pharmacokinetic analyses were not included in the trial design of PIONEER 12 so it is not possible to confirm this hypothesis. It is important to note that PIONEER 3 was a larger trial than PIONEER 12 (1864 participants vs 1441 participants, respectively) and had a longer duration (78 weeks with 5 weeks of follow-up vs 26 weeks with 5 weeks of follow-up, respectively) [18]. Because the incidence of AEs was greatest during dose escalation, the disparity in total years of exposure between the two trials could potentially dilute the reported rate of AEs in PIONEER 3 compared with PIONEER 12. Consistent with the previous global PIONEER trials, few SAEs were reported across treatment arms and most were reported in the oral semaglutide 3 mg and sitagliptin 100 mg treatment arms. Overall, the safety and tolerability profile for oral semaglutide in PIONEER 12 was consistent with the results from the global PIONEER trials and with the general safety profile of the GLP-1RA drug class [27].

The strengths of this trial include the high number of participants enrolled and randomised, particularly in the Chinese subpopulation, and the masking of differences in the appearance of the drugs. Potential limitations of this trial include the short trial duration. At 26 weeks, PIONEER 12 was relatively short compared with most of the global PIONEER trials, except for PIONEER 1 and 5, and was considerably shorter than the 78 week PIONEER 3 trial that it mirrors. However, considering that the safety and tolerability profile of oral semaglutide in PIONEER 12 was consistent with that of the global PIONEER trials and with the safety profile of the GLP-1RA drug class, it is reasonable to assume that long-term safety in this population may be consistent with that seen in the long-term global trials. Another limitation is that no specific analyses were performed regarding the impact of sex, gender, race or ethnicity on the results of this trial. As such, caution should be taken when applying the results to a wider population. In addition, treatment adherence was not formally measured and participants were instead reminded by the trial investigators to follow the treatment protocol at each visit, with the investigators also monitoring drug accountability. A final limitation is the use of sitagliptin as a comparator. DPP-4is have modest glucose-lowering effects and minimal body weight loss effects compared with GLP-1RAs [24]; however, their use is increasing steadily in Chinese populations [28].

In conclusion, this trial demonstrates the superiority of oral semaglutide vs sitagliptin in reducing HbA_1c_ and body weight from baseline in a predominantly Chinese population with type 2 diabetes, as well as a safety and tolerability profile for semaglutide that is consistent with that of the GLP-1RA class.

## Supplementary Information

Below is the link to the electronic supplementary material.

Supplementary file1 (PDF 463 KB)

## Data Availability

Data will be shared with bona fide researchers submitting a research proposal approved by the independent review board. Information on requesting access to datasets can be found at www.novonordisk-trials.com. Data will be made available after research completion and approval of the product and product use in the European Union and the USA. Individual participant data will be shared in data sets in a de-identified/anonymised format.

## Abbreviations

AACE: American Association of Clinical Endocrinologists
AE: Adverse event
CDS: Chinese Diabetes Society
DPP-4i: Dipeptidyl peptidase-4 inhibitor
ETD: Estimated treatment difference
FAS: Full analysis set
FPG: Fasting plasma glucose
GLP-1RA: Glucagon-like peptide-1 receptor agonist
PIONEER: Peptide Innovation for Early Diabetes Treatment
SAE: Serious adverse event
SAS: Safety analysis set
SF-36v2: 36-item Short Form Health Survey (Acute Version)
SMPG: Self-monitored plasma glucose

## Authors’ relationships and activities

LJ reports receiving consulting and lecture fees from Abbott, AstraZeneca, Bayer, Boehringer Ingelheim, Eli Lilly, Medtronic, Merck, MSD, Novo Nordisk, Roche and Sanofi-Aventis. RMA and SG are employees and shareholders of Novo Nordisk A/S. SCB has received honoraria, teaching and research sponsorship/grants from AstraZeneca, Boehringer Ingelheim, Bristol Myers Squibb, Eli Lilly, GlaxoSmithKline, MSD, Novo Nordisk, Pfizer, Sanofi and Takeda. BL and YT are employees of Novo Nordisk (China) Pharmaceuticals Co. All other authors declare that there are no relationships or activities that might bias, or be perceived to bias, their work.
