# Supplementary material for: Efficacy and safety of oral semaglutide vs sitagliptin in a predominantly Chinese population with type 2 diabetes uncontrolled with metformin: PIONEER 12, a double-blind, Phase IIIa, randomised trial
Source: Diabetologia. 2024 Jul 10;67(9):1800–16. doi: 10.1007/s00125-024-06133-4 (PMC11410852; doi:10.1007/s00125-024-06133-4)
Supplement: Supplementary file 1 — Supplementary file1 (PDF 463 KB) [file 125_2024_6133_MOESM1_ESM.pdf]

# Efficacy and safety of oral semaglutide vs sitagliptin in a predominantly Chinese population with type 2 diabetes uncontrolled with metformin: PIONEER 12, a double-blind, phase IIIa, randomised trial

Linong Ji,[1] Rikke M. Agesen,[2] Stephen C. Bain,[3] Fangming Fu,[4] Sanaz Gabery,[2] Jianlin Geng,[5] Yiming Li,[6] Yibing Lu,[7] Bifen Luo,[8] Wuyan Pang,[9] Yi Tao[8], for the PIONEER 12 investigators

[1]Peking University People's Hospital, Beijing, China

[2]Novo Nordisk A/S, Søborg, Denmark

[3]Diabetes Research Unit, Swansea University, Swansea, UK

[4]Jinan Central Hospital Affiliated to Shandong First Medical University, Jinan, Shandong Province, China

[5]Harrison International Peace Hospital, Hengshui, China

[6]Huashan Hospital, Fudan University, Shanghai, China

[7]Second Affiliated Hospital of Nanjing Medical University, Nanjing, China

[8]Novo Nordisk (China) Pharmaceuticals Co., Ltd., Beijing, China

[9]Huaihe Hospital of Henan University, Kaifeng, Henan, China

**ESM Appendix 1** PIONEER 12 investigators

Xiaolin Dong,[1] Wuyan Pang,[2] Jianlin Geng,[3] Fang Bian,[4] Yibing Lu,[5] Shu Li,[6] Guoyue Yuna,[7] Ji Hu,[8] Liujun Fu,[9] Yufeng Li,[10] Xiaozhen Jiang,[11] Shan Huang,[12] Keqin Zhang,[13] Xinhua Ye,[14] Jun Liu,[15] Yiming Li,[16] Ying Zhang,[17] Nannan Yu,[18] Bimin Shi,[19] Shandong Ye,[20] Hongwei Ling,[21] Qiu Zhang,[22] Ming Liu,[23] Yongqian Liang,[24] Xuefeng Li,[25] Ruifang Bu,[26] Yongli Yao,[27] Huibin Huang,[28] Lihui Zhang,[29] Wenshan Lv,[30] Fenglin Cao,[31] Weihong Song,[32] Youping Dong,[33] Bo Feng,[34] Shen Qu,[35] Mei Lin,[36] Hanqing Cai,[37] Qi Xu,[38] Gangyi Yang,[39] Chaohui Mo,[40] Kun Wang,[41] Xiaohong Jiang,[42] Yonghong Zhang,[43] Dong Zhao,[44] Zhihong Wang,[45] Yu Liu,[46] Xinhua Xiao,[47] Mingtong Xu,[48] Wenbo Wang,[49] Huige Shao,[50] Linong Ji,[51] Yao Weng,[52] Jing Xu,[53] Yawei Zhang,[54] Qing Wang,[55] Yushan Xu,[56] Yinghong Du,[57] Yuezhong Ren.[58]

[1]Jinan Central Hospital Affiliated to Shandong University, Jinan, China; [2]Huaihe Hospital of Henan University, Kaifeng, China; [3]Harrison International Peace Hospital, Hengshui, China; [4]Cangzhou People’s Hospital, Cangzhou, China; [5]The Second Affiliated Hospital of Nanjing Medical University, Nanjing, China; [6]Huizhou Central People’s Hospital, Huizhou, China; [7]The Affiliated Hospital of Jiangsu University, Zhenjiang, China; [8]The Second Affiliated Hospital of Soochow University, Suzhou, China; [9]The First Affiliated Hospital of Henan University of Science and Technology, Luoyang, China; [10]Beijing Pinggu Hospital, Beijing, China; [11]Shanghai Pudong New District People’s Hospital, Shanghai, China; [12]Shanghai Tongren Hospital, Shanghai, China; [13]Tongji Hospital of Tongji University, Shanghai, China; [14]Changzhou No.2 People’s Hospital, Changzhou, China; [15]The Fifth People’s Hospital of Shanghai, Fudan University, Shanghai, China; [16]Huashan Hospital Affiliated to Fudan University, Shanghai, China; [17]Jilin Province People’s Hospital, Changchun, China; [18]The 2nd Affiliated Hospital of Kunming Medical University, Kunming, China; [19]The First Affiliated Hospital of Soochow University, Suzhou, China; [20]Anhui Provincial Hospital, Hefei, China; [21]Affiliated Hospital of Xuzhou Medical University, Xuzhou, China; [22]The First Affiliated Hospital of Anhui Medical University, Hefei, China; [23]Tianjin Medical University General

Hospital, Tianjin, China; [24]Shunde Hospital of Southern Medical University, Shunde, China; [25]Taihe Hospital, Shiyan, China; [26]Wuxi People’s Hospital, Wuxi, China; [27]Qinghai Provincial Hospital, Xining, China; [28]The Second Affiliated Hospital of Fujian Medical University, Quanzhou, China; [29]The Second Hospital of Hebei Medical University, Shijiazhuang, China; [30]Affiliated Hospital of Qingdao University, Qingdao, China; [31]The Second Hospital of Tianjin Medical University, Tianjin, China; [32]Chenzhou No.1 People’s Hospital, Chenzhou, China; [33]General Hospital of Ningxia Medical University, Yinchuan, China; [34]Dongfang Hospital Affiliated to Shanghai Tongji University, Shanghai, China; [35]Shanghai Tenth People’s Hospital, Shanghai, China; [36]Wuhan Puai Hospital, Wuhan, China; [37]The Second Hospital of Jilin University, Changchun, China; [38]The Second Affiliated Hospital of Shantou University Medical College, Shantou, China; [39]The Second Affiliated Hospital of Chongqing Medical University, Chongqing, China; [40]The Third Xiangya Hospital of Central South University, Changsha, China; [41]Nanjing Jiangning Hospital, Nanjing, China; [42]The First People’s Hospital of Changzhou, Changzhou, China; [43]The First People’s Hospital of Yunnan Province, Kunming, China; [44]Beijing Luhe Hospital Capital Medical University, Beijing, China; [45]Zhengzhou First People’s Hospital, Zhengzhou, China; [46]Sir Run Run Hospital Nanjing Medical University, Nanjing, China; [47]The First Affiliated Hospital of University of South China, Hengyang, China; [48]Sun Yat-sen Memorial Hospital, Sun Yat-sen University, Guangzhou, China; [49]Peking University Shougang Hospital, Beijing, China; [50]Changsha Central Hospital, Changsha, China; [51]Peking University People’s Hospital, Beijing, China; [52]Zhuzhou Central Hospital, Zhuzhou, China; [53]The Second Affiliated Hospital of Xi’an Jiaotong University, Xi'an, China; [54]PingXiang People’s Hospital, Pingxiang, China; [55]China-Japan Union Hospital of Jilin University, Changchun, China; [56]First Affiliated Hospital of Kunming Medical University, Kunming, China; [57]Guangzhou Panyu Central Hospital, Guangzhou, China; [58]The Second Affiliated Hospital of Zhejiang University School of Medicine, Hangzhou, China.

**ESM Appendix 2** Impact of COVID-19 summary

Adherence to the trial protocol was maintained where possible during the global COVID-19 pandemic. Planned physical visits could take place outside of the protocol-defined window or be converted to phone or video contacts to ensure participant safety. If the end-of-trial visit (visit 8) could not be attended, additional trial product could be raised for a further 3–5 weeks, with primary endpoint data to be collected to the extent possible within the expanded visit 8 window. When participants could not attend site visits, safety laboratory assessments could be performed at a local laboratory at the investigators' discretion. Any deviations in trial protocol due to COVID-19 were evaluated.

**ESM Appendix 3** Methods

**Statistical analysis**

The following series of observation periods was defined: (1) the in-trial observation period represented the period where participants were considered in the trial, regardless of trial product discontinuation or rescue medication, ranging from randomisation until either the follow up visit, participant withdrawal or death; (2) the on-treatment observation period represented the period where participants were considered treated with the trial product following randomisation; (3) the on-treatment without rescue medication period was a subset of the on-treatment period, where participants were considered treated with trial product but had not initiated any rescue medication, ranging from trial product start until either the last dose of trial product plus 3 days or initiation of rescue medication.

For the primary analysis of the treatment policy estimand, a pattern mixture model using multiple imputation to handle missing data was used. Change from baseline at week 26 in $HbA_{1c}$ was analysed using analysis of covariance, with treatment and region as categorical fixed effects and baseline $HbA_{1c}$ as covariate.

**ESM Table 1** Inclusion and exclusion criteria

| Inclusion criteria |
|---|
| 1. Informed consent obtained before any trial-related activities. Trial-related activities are defined as any procedures that are carried out as part of the trial, including activities to determine suitability for the trials. |
| 2. Male or female, aged ≥18 years at the time of signing informed consent.<br>*For Taiwan only: male or female, aged ≥20 years at the time of signing informed consent.* |
| 3. Diagnosed with type 2 diabetes ≥60 days prior to day of screening. |
| 4. $HbA_{1c}$ 53–91 mmol/mol (7.0–10.5%) (both inclusive). |
| 5. Stable daily dose of metformin (≥1500 mg or maximum tolerated dose as documented in the participant medical record) ≥60 days prior to day of screening. |
| **Exclusion criteria** |
| 1. Known or suspected hypersensitivity to trial products or related products. |
| 2. Previous participation in this trial. Participation is defined as signed informed consent. |
| 3. Person who is pregnant, breast-feeding or intends to become pregnant, or is of child-bearing potential and not using a highly effective contraceptive method. |
| 4. Receipt of any investigational medicinal product within 90 days before screening. |
| 5. Any disorder which in the investigator’s opinion might jeopardise participant safety or compliance with the protocol. |
| 6. Family or personal history of multiple endocrine neoplasia type 2 or medullary thyroid carcinoma. Family is defined as a first-degree relative. |
| 7. History or presence of pancreatitis (acute or chronic). |
| 8. History of major surgical procedures involving the stomach, potentially affecting absorption of trial product (e.g. subtotal and total gastrectomy, sleeve gastrectomy, gastric bypass surgery). |
| 9. Any of the following: myocardial infarction, stroke, or hospitalisation for unstable angina or transient ischaemic attack within the past 180 days prior to the day of screening and randomisation. |
| 10. Participants presently classified as being in New York Heart Association Class IV. |
| 11. Planned coronary, carotid or peripheral artery revascularisation known on the day of screening. |
| 12. Renal impairment measured as estimated glomerular filtration rate <60 ml/min per 1.73 $m^2$ as per Chronic Kidney Disease Epidemiology Collaboration formula. |

13. Participants with alanine aminotransferase >2.5 × upper limit of the normal.
14. Treatment with once-weekly glucagon-like peptide-1 receptor agonist (GLP-1RA) or thiazolidinedione within the past 90 days prior to the day of screening.
15. Treatment with any medication for the indication of diabetes or obesity other than stated in the inclusion criteria within the past 60 days prior to the day of screening. However, short-term insulin treatment for a maximum of 14 days prior to the day of screening is allowed.
16. Use of non-herbal Chinese medicine or other non-herbal local medicine with unknown or unspecified content. Herbal traditional Chinese medicine or other local herbal medicines may, at the investigator’s discretion, be continued throughout the trial
17. Uncontrolled and potentially unstable diabetic retinopathy or maculopathy, verified by a fundus examination performed within the past 90 days prior to screening or in the period between screening and randomisation. Pharmacological pupil-dilation is a requirement unless using a digital fundus photography camera specified for nondilated examination
18. Presence or history of malignant neoplasms within the past 5 years prior to the day of screening. Basal and squamous cell skin cancer and any carcinoma in-situ is allowed.

**ESM Table 2** Additional supportive secondary endpoints

| Endpoints | Trial product estimand[a] | | | | Treatment policy estimand[b] | | | |
|---|---|---|---|---|---|---|---|---|
| | Oral semaglutide | | | Sitagliptin | Oral semaglutide | | | Sitagliptin |
| | 3 mg | 7 mg | 14 mg | 100 mg | 3 mg | 7 mg | 14 mg | 100 mg |
| *N* | 361 | 360 | 361 | 359 | 361 | 360 | 361 | 359 |
| SF-36v2 – general health norm-based score | | | | | | | | |
| *n* | 322 | 320 | 299 | 334 | 343 | 343 | 332 | 350 |
| Estimated mean | 50.6 | 50.6 | 51.2 | 50.5 | 50.4 | 50.1 | 50.9 | 50.5 |
| Change from baseline | 1.2 | 1.3 | 1.9 | 1.2 | 1.0 | 0.7 | 1.6 | 1.2 |
| ETD vs sitagliptin (95% CI) | 0.1 (–1.0, 1.1) | 0.1 (–0.9, 1.1) | 0.7 (–0.3, 1.7) | – | –0.2 (–1.2, 0.9) | –0.5 (–1.5, 0.6) | 0.4 (–0.7, 1.4) | – |
| *p* value | 0.9225 | 0.8395 | 0.1817 | – | 0.7632 | 0.3640 | 0.4972 | – |
| SF-36v2 – mental health norm-based score | | | | | | | | |
| *n* | 322 | 320 | 299 | 334 | 343 | 343 | 332 | 350 |
| Estimated mean | 53.2 | 53.7 | 53.6 | 52.6 | 53.0 | 53.4 | 53.4 | 52.5 |

| Endpoints | Trial product estimand[a] | | | | Treatment policy estimand[b] | | | |
|---|---|---|---|---|---|---|---|---|
| | Oral semaglutide | | | Sitagliptin | Oral semaglutide | | | Sitagliptin |
| | 3 mg | 7 mg | 14 mg | 100 mg | 3 mg | 7 mg | 14 mg | 100 mg |
| Change from baseline | 0.1 | 0.6 | 0.5 | –0.4 | –0.1 | 0.3 | 0.3 | –0.5 |
| ETD vs sitagliptin (95% CI) | 0.6 (–0.4, 1.5) | 1.0 (0.1, 2.0) | 0.9 (–0.0, 1.9) | – | 0.5 (–0.6, 1.5) | 0.8 (–0.2, 1.9) | 0.8 (–0.2, 1.9) | – |
| *p* value | 0.2569 | 0.0337 | 0.0577 | – | 0.3934 | 0.1108 | 0.1297 | – |
| BMI at week 26, kg/m$^2$ | | | | | | | | |
| *n* | 322 | 319 | 300 | 335 | 343 | 342 | 333 | 351 |
| Estimated mean | 28.1 | 27.5 | 27.2 | 28.4 | 28.1 | 27.6 | 27.3 | 28.4 |
| Change from baseline | –0.1 | –1.0 | –1.4 | –0.2 | –0.5 | –1.0 | –1.3 | –0.2 |
| ETD vs sitagliptin (95% CI) | –0.3 (–0.5, –0.1) | –0.8 (–1.0, –0.7) | –1.2 (–1.4, –1.0) | – | –0.3 (–0.5, –0.2) | –0.8 (–1.0, –0.6) | –1.1 (–1.3, –0.9) | – |
| *p* value | 0.0003 | <0.0001 | <0.0001 | – | 0.0002 | <0.0001 | <0.0001 | – |

| Endpoints | Trial product estimand[a] | | | | Treatment policy estimand[b] | | | |
|---|---|---|---|---|---|---|---|---|
| | Oral semaglutide | | | Sitagliptin | Oral semaglutide | | | Sitagliptin |
| | 3 mg | 7 mg | 14 mg | 100 mg | 3 mg | 7 mg | 14 mg | 100 mg |
| Waist circumference at week 26, cm | | | | | | | | |
| *n* | 322 | 320 | 300 | 335 | 343 | 343 | 333 | 351 |
| Estimated mean | 96.5 | 95.3 | 94.2 | 97.2 | 96.2 | 95.2 | 94.3 | 96.9 |
| Change from baseline | –1.5 | –2.7 | –3.8 | –0.8 | –1.5 | –2.5 | –3.4 | –0.8 |
| ETD vs sitagliptin (95% CI) | –0.7 (–1.3, –0.1) | –1.8 (–2.4, –1.2) | –2.9 (–3.5, –2.3) | – | –0.7 (–1.3, –0.1) | –1.7 (–2.3, –1.1) | –2.5 (–3.1, –2.0) | – |
| *p* value | 0.0254 | <0.0001 | <0.0001 | – | 0.0200 | <0.0001 | <0.0001 | – |
| 7-point self-monitored blood glucose postprandial increment, mmol/l | | | | | | | | |
| *n* | 301 | 311 | 290 | 317 | 320 | 333 | 318 | 333 |
| Estimated mean | 2.4 | 2.0 | 1.9 | 2.3 | 2.5 | 2.1 | 2.0 | 2.3 |
| Change from baseline | –0.5 | –0.9 | –1.0 | –0.6 | –0.4 | –0.8 | –0.9 | –0.6 |

| Endpoints | Trial product estimand[a] | | | | Treatment policy estimand[b] | | | |
|---|---|---|---|---|---|---|---|---|
| | Oral semaglutide | | | Sitagliptin | Oral semaglutide | | | Sitagliptin |
| | 3 mg | 7 mg | 14 mg | 100 mg | 3 mg | 7 mg | 14 mg | 100 mg |
| ETD vs sitagliptin (95% CI) | 0.1 (–0.1, 0.3) | –0.3 (–0.6, –0.1) | –0.4 (–0.6, –0.2) | – | 0.2 (–0.1, 0.4) | –0.2 (–0.5, –0.0) | –0.3 (–0.6, –0.1) | – |
| *p* value | 0.3917 | 0.0051 | 0.0009 | – | 0.1920 | 0.0428 | 0.0056 | – |
| 7-point self-monitored blood glucose postprandial increment, mg/dl | | | | | | | | |
| *n* | 301 | 311 | 290 | 317 | 320 | 333 | 318 | 333 |
| Estimated mean | 44.0 | 36.2 | 34.9 | 42.1 | 44.7 | 37.4 | 35.6 | 41.8 |
| Change from baseline | –8.5 | –16.3 | –17.6 | –10.4 | –7.7 | –15.0 | –16.8 | –10.6 |
| ETD vs sitagliptin (95% CI) | 1.8 (–2.4, 6.0) | –5.9 (–10.1, –1.8) | –7.2 (–11.4, –3.0) | – | 2.9 (–1.5, 7.3) | –4.4 (–8.7, –0.1) | –6.3 (–10.7, –1.8) | – |
| *p* value | 0.3916 | 0.0051 | 0.0009 | – | 0.1920 | 0.0428 | 0.0056 | – |

AACE, American Association of Clinical Endocrinologists; ETD, estimated treatment difference; *N*, number of participants contributing to the analysis; *n*, number of participants with an observation at the visit; SF-36v2, 36-item Short Form Health Survey (Acute Version)

**ESM Table 3** Additional concomitant anti-hyperglycaemic medication including rescue medication and time to rescue medication

| | Oral semaglutide 3 mg (*N*=361) | Oral semaglutide 7 mg (*N*=360) | Oral semaglutide 14 mg (*N*=361) | Sitagliptin 100 mg (*N*=359) | Total (*N*=1441) |
|---|---|---|---|---|---|
| Number of participants on additional concomitant anti-hyperglycaemic medication, *n* (%) | 14 (3.9) | 11 (3.1) | 17 (4.7) | 14 (3.9) | 56 (3.9) |
| Number of participants on rescue medication, *n* (%) | 5 (1.4) | 6 (1.7) | 6 (1.7) | 7 (1.9) | 24 (1.7) |
| Sulfonylureas | 2 (0.6) | 3 (0.8) | 2 (0.6) | 4 (1.1) | 11 (0.8) |
| Long-acting insulin | 0 | 3 (0.8) | 1 (0.3) | 1 (0.3) | 5 (0.3) |
| Alpha glucosidase inhibitors | 3 (0.8) | 1 (0.3) | 0 | 0 | 4 (0.3) |
| Sodium-glucose co-transporter 2 inhibitors | 0 | 0 | 2 (0.6) | 1 (0.3) | 3 (0.2) |
| Intermediate- or long-acting combined with fast-acting insulin | 0 | 0 | 2 (0.6) | 0 | 2 (0.1) |
| Biguanides | 1 (0.3) | 0 | 0 | 1 (0.3) | 2 (0.1) |
| Intermediate-acting insulin | 1 (0.3) | 0 | 0 | 0 | 1 (0.1) |

| Time from first dose to rescue medication with oral semaglutide vs sitagliptin 100 mg[a] | | |
|---|---|---|
| | HR (95% CI) | *p* value |
| 3 mg | 0.85 (0.27, 2.69) | 0.7781 |
| 7 mg | 1.01 (0.34, 3.02) | 0.9853 |
| 14 mg | 1.10 (0.37, 3.30) | 0.8657 |

Additional concomitant anti-hyperglycaemic medication describes new and/or intensified anti-hyperglycaemic medication initiated at or after randomisation and before the planned end-of-treatment. Rescue medication is a subset of additional concomitant anti-hyperglycaemic medication and was prescribed at investigator's discretion as add-on to trial product according to ADA/EASD guidelines. GLP-1RAs, DPP-4is and amylin analogues were not allowed as rescue medicines.

[a]Data were from the on-treatment without rescue medication period. Time to initiation of rescue medication was analysed using a Cox proportional hazards model, with treatment and region as categorical fixed effects and baseline $HbA_{1c}$ as covariate. Censoring time was 1 day before last day on trial product

**ESM Table 4** External event adjudication committee-confirmed events and selected in-trial adverse events

| | Oral semaglutide | | | Sitagliptin 100 mg (*N*=358) |
|---|---|---|---|---|
| | 3 mg (*N*=361) | 7 mg (*N*=358) | 14 mg (*N*=361) | |
| Death | 2 (0.6) | 2 (0.6) | 1 (0.3) | 0 |
| Cardiovascular death | 0 | 1 (0.3) | 0 | 0 |
| Undetermined cause of death | 0 | 0 | 1 (0.3) | 0 |
| Non-cardiovascular cause of death | 2 (0.6) | 1 (0.3) | 0 | 0 |
| Acute kidney injury | 1 (0.3) | 0 | 2 (0.6) | 1 (0.3) |
| Cardiovascular events | 1 (0.3) | 3 (0.8) | 5 (1.4) | 2 (0.6) |
| Acute coronary syndrome | 1 (0.3) | 1 (0.3) | 0 | 1 (0.3) |
| Acute myocardial infarction | 1 (0.3) | 1 (0.3) | 0 | 1 (0.3) |
| Cerebrovascular events | 0 | 1 (0.3) | 3 (0.8) | 1 (0.3) |
| Stroke | 0 | 1 (0.3) | 3 (0.8) | 1 (0.3) |
| Cardiovascular and undetermined cause of death | 0 | 1 (0.3) | 1 (0.3) | 0 |
| Cardiovascular death | 0 | 1 (0.3) | 0 | 0 |
| Undetermined cause of death | 0 | 0 | 1 (0.3) | 0 |
| Heart failure | 0 | 0 | 1 (0.3) | 0 |
| Malignant neoplasm[a] | 0 | 0 | 1 (0.3) | 0 |
| Lung and pleura cancer | 0 | 0 | 1 (0.3) | 0 |

Data are *n* (%)

[a]Excludes malignant thyroid neoplasms

**ESM Table 5** Vital signs and laboratory safety assessments (safety analysis set)

| | In-trial observation period | | | | On-treatment observation period | | | |
|---|---|---|---|---|---|---|---|---|
| | Oral semaglutide | | | Sitagliptin | Oral semaglutide | | | Sitagliptin |
| | 3 mg | 7 mg | 14 mg | 100 mg | 3 mg | 7 mg | 14 mg | 100 mg |
| Pulse rate at week 26, beats/min | | | | | | | | |
| *n* | 342 | 343 | 333 | 350 | 325 | 325 | 305 | 340 |
| Estimated mean | 79 | 80 | 81 | 78 | 79 | 81 | 82 | 78 |
| Change from baseline | 1 | 2 | 3 | 0 | 1 | 3 | 4 | 0 |
| ETD vs sitagliptin (95% CI) | 1 (–0, 2) | 2 (1, 3) | 3 (1, 4) | – | 1 (–0, 2) | 2 (1, 3) | 4 (2, 5) | – |
| *p* value | 0.1186 | 0.0021 | <0.0001 | – | 0.0959 | 0.0003 | <0.0001 | – |
| Systolic blood pressure at week 26, mmHg | | | | | | | | |
| *n* | 342 | 343 | 333 | 350 | 325 | 325 | 305 | 340 |
| Estimated mean | 129 | 127 | 128 | 129 | 129 | 127 | 128 | 129 |
| Change from baseline | –2 | –3 | –3 | –2 | –2 | –4 | –3 | –2 |

| | In-trial observation period | | | | On-treatment observation period | | | |
|---|---|---|---|---|---|---|---|---|
| | Oral semaglutide | | | Sitagliptin | Oral semaglutide | | | Sitagliptin |
| | 3 mg | 7 mg | 14 mg | 100 mg | 3 mg | 7 mg | 14 mg | 100 mg |
| ETD vs sitagliptin (95% CI) | 1 (–1, 2) | –1 (–3, 1) | –1 (–2, 1) | – | 0 (–1, 2) | –2 (–3, –0) | –1 (–3, 0) | – |
| *p* value | 0.4465 | 0.2436 | 0.4150 | – | 0.6725 | 0.0418 | 0.1586 | – |
| Diastolic blood pressure at week 26, mmHg | | | | | | | | |
| *n* | 342 | 343 | 333 | 350 | 325 | 325 | 305 | 340 |
| Estimated mean | 83 | 82 | 83 | 82 | 83 | 82 | 83 | 82 |
| Change from baseline | 0 | –1 | 0 | –1 | 0 | –1 | 0 | –1 |
| ETD vs sitagliptin (95% CI) | 1 (–0, 2) | 1 (–0, 2) | 1 (–0, 2) | – | 1 (–0, 2) | 1 (–1, 2) | 1 (–0, 2) | – |
| *p* value | 0.0760 | 0.1524 | 0.0717 | – | 0.1239 | 0.3016 | 0.0809 | – |
| Estimated glomerular filtration rate[a] ratio to baseline at week 26 | | | | | | | | |
| *n* | 340 | 343 | 331 | 349 | 323 | 324 | 304 | 338 |

| | In-trial observation period | | | | On-treatment observation period | | | |
|---|---|---|---|---|---|---|---|---|
| | Oral semaglutide | | | Sitagliptin | Oral semaglutide | | | Sitagliptin |
| | 3 mg | 7 mg | 14 mg | 100 mg | 3 mg | 7 mg | 14 mg | 100 mg |
| Geometric mean (CV) | 1.0 (8.6) | 1.0 (8.5) | 1.0 (7.8) | 1.0 (7.4) | 1.0 (8.7) | 1.0 (8.4) | 1.0 (7.8) | 1.0 (7.3) |
| Calcitonin at week 26, ng/l | | | | | | | | |
| *n* | 344 | 344 | 331 | 349 | 326 | 325 | 305 | 338 |
| Geometric mean (CV) | 1.4 (77.9) | 1.4 (74.6) | 1.4 (85.2) | 1.3 (64.5) | 1.4 (78.9) | 1.4 (75.9) | 1.5 (87.4) | 1.3 (63.4) |
| Alanine aminotransferase at week 26, U/l | | | | | | | | |
| *n* | 340 | 341 | 331 | 346 | 323 | 322 | 304 | 335 |
| Geometric mean (CV) | 22 (60) | 21 (66) | 20 (56) | 24 (64) | 22 (60) | 22 (66) | 20 (57) | 24 (63) |
| Aspartate aminotransferase at week 26, U/l | | | | | | | | |
| *n* | 340 | 341 | 331 | 344 | 323 | 322 | 304 | 334 |
| Geometric mean (CV) | 19 (38) | 19 (43) | 18 (34) | 20 (44) | 19 (39) | 19 (43) | 18 (35) | 20 (45) |

| | In-trial observation period | | | | On-treatment observation period | | | |
|---|---|---|---|---|---|---|---|---|
| | Oral semaglutide | | | Sitagliptin | Oral semaglutide | | | Sitagliptin |
| | 3 mg | 7 mg | 14 mg | 100 mg | 3 mg | 7 mg | 14 mg | 100 mg |
| Alkaline phosphatase at week 26, U/l | | | | | | | | |
| *n* | 340 | 343 | 331 | 349 | 323 | 324 | 304 | 338 |
| Geometric mean (CV) | 70 (30) | 70 (31) | 69 (28) | 69 (28) | 70 (29) | 70 (32) | 69 (28) | 69 (29) |
| Total bilirubin at week 26, µmol/l | | | | | | | | |
| *n* | 340 | 341 | 331 | 346 | 323 | 322 | 304 | 335 |
| Geometric mean (CV) | 10.6 (50.0) | 9.8 (45.6) | 10.5 (46.9) | 10.1 (48.3) | 10.6 (50.6) | 9.9 (46.0) | 10.5 (47.4) | 10.0 (47.4) |
| Total bilirubin at week 26, mg/dl | | | | | | | | |
| *n* | 340 | 341 | 331 | 346 | 323 | 322 | 304 | 335 |
| Geometric mean (CV) | 0.6 (50.0) | 0.6 (45.6) | 0.6 (47.0) | 0.6 (48.3) | 0.6 (50.6) | 0.6 (46.0) | 0.6 (47.4) | 0.6 (47.4) |
| Creatinine at week 26, µmol/l | | | | | | | | |
| *n* | 340 | 343 | 331 | 349 | 323 | 324 | 304 | 338 |

| | In-trial observation period | | | | On-treatment observation period | | | |
|---|---|---|---|---|---|---|---|---|
| | Oral semaglutide | | | Sitagliptin | Oral semaglutide | | | Sitagliptin |
| | 3 mg | 7 mg | 14 mg | 100 mg | 3 mg | 7 mg | 14 mg | 100 mg |
| Geometric mean (CV) | 62.9 (23.7) | 64.2 (23.4) | 62.9 (22.8) | 61.1 (23.2) | 62.9 (23.9) | 64.0 (23.4) | 63.0 (22.5) | 60.9 (22.9) |
| Creatinine at week 26, mg/dl | | | | | | | | |
| *n* | 340 | 343 | 331 | 349 | 323 | 324 | 304 | 338 |
| Geometric mean (CV) | 0.7 (23.7) | 0.7 (23.4) | 0.7 (22.8) | 0.7 (23.2) | 0.7 (23.9) | 0.7 (23.4) | 0.7 (22.5) | 0.7 (22.9) |
| Albumin at week 26, g/dl | | | | | | | | |
| *n* | 340 | 343 | 331 | 349 | 323 | 324 | 304 | 338 |
| Geometric mean (CV) | 4.5 (5.4) | 4.5 (6.0) | 4.5 (5.7) | 4.5 (6.1) | 4.5 (5.4) | 4.5 (6.1) | 4.5 (5.7) | 4.5 (6.1) |
| Urea at week 26, mmol/l | | | | | | | | |
| *n* | 340 | 343 | 331 | 349 | 323 | 324 | 304 | 338 |
| Geometric mean (CV) | 5.1 (28.2) | 5.0 (28.5) | 5.1 (27.2) | 5.0 (28.1) | 5.1 (28.5) | 5.0 (28.6) | 5.0 (26.8) | 4.9 (27.8) |

| | In-trial observation period | | | | On-treatment observation period | | | |
|---|---|---|---|---|---|---|---|---|
| | Oral semaglutide | | | Sitagliptin | Oral semaglutide | | | Sitagliptin |
| | 3 mg | 7 mg | 14 mg | 100 mg | 3 mg | 7 mg | 14 mg | 100 mg |
| Calcium at week 26, mmol/l | | | | | | | | |
| *n* | 340 | 343 | 331 | 349 | 323 | 324 | 304 | 338 |
| Geometric mean (CV) | 2.3 (4.1) | 2.3 (4.5) | 2.3 (4.7) | 2.3 (4.8) | 2.3 (4.1) | 2.3 (4.5) | 2.3 (4.7) | 2.3 (4.8) |
| Calcium at week 26, mg/dl | | | | | | | | |
| *n* | 340 | 343 | 331 | 349 | 323 | 324 | 304 | 338 |
| Geometric mean (CV) | 9.3 (4.1) | 9.3 (4.5) | 9.3 (4.7) | 9.2 (4.8) | 9.3 (4.1) | 9.3 (4.5) | 9.3 (4.7) | 9.2 (4.8) |
| Potassium at week 26, mmol/l | | | | | | | | |
| *n* | 340 | 343 | 331 | 348 | 323 | 324 | 304 | 337 |
| Geometric mean (CV) | 4.3 (8.2) | 4.3 (9.1) | 4.3 (8.8) | 4.3 (9.0) | 4.3 (8.1) | 4.3 (9.0) | 4.3 (8.7) | 4.3 (8.9) |
| Potassium at week 26, mEq/l | | | | | | | | |
| *n* | 340 | 343 | 331 | 348 | 323 | 324 | 304 | 337 |

| | In-trial observation period | | | | On-treatment observation period | | | |
|---|---|---|---|---|---|---|---|---|
| | Oral semaglutide | | | Sitagliptin | Oral semaglutide | | | Sitagliptin |
| | 3 mg | 7 mg | 14 mg | 100 mg | 3 mg | 7 mg | 14 mg | 100 mg |
| Geometric mean (CV) | 4.3 (8.2) | 4.3 (9.1) | 4.3 (8.8) | 4.3 (9.0) | 4.3 (8.1) | 4.3 (9.0) | 4.3 (8.7) | 4.3 (8.9) |
| Sodium at week 26, mmol/l | | | | | | | | |
| *n* | 340 | 343 | 331 | 348 | 323 | 324 | 304 | 337 |
| Geometric mean (CV) | 140 (1) | 140 (1) | 140 (1) | 140 (2) | 140 (1) | 140 (1) | 140 (1) | 140 (2) |
| Sodium at week 26, mEq/l | | | | | | | | |
| *n* | 340 | 343 | 331 | 348 | 323 | 324 | 304 | 337 |
| Geometric mean (CV) | 140 (1) | 140 (1) | 140 (1) | 140 (2) | 140 (1) | 140 (1) | 140 (1) | 140 (2) |

[a]Glomerular filtration rate was estimated by the CKD-EPI formula

CKD-EPI, Chronic Kidney Disease Epidemiology Collaboration; ETD, estimated treatment difference; *n*, number of participants with an observation at the visit; SAS, safety analysis set

ESM Fig. 1 Trial design

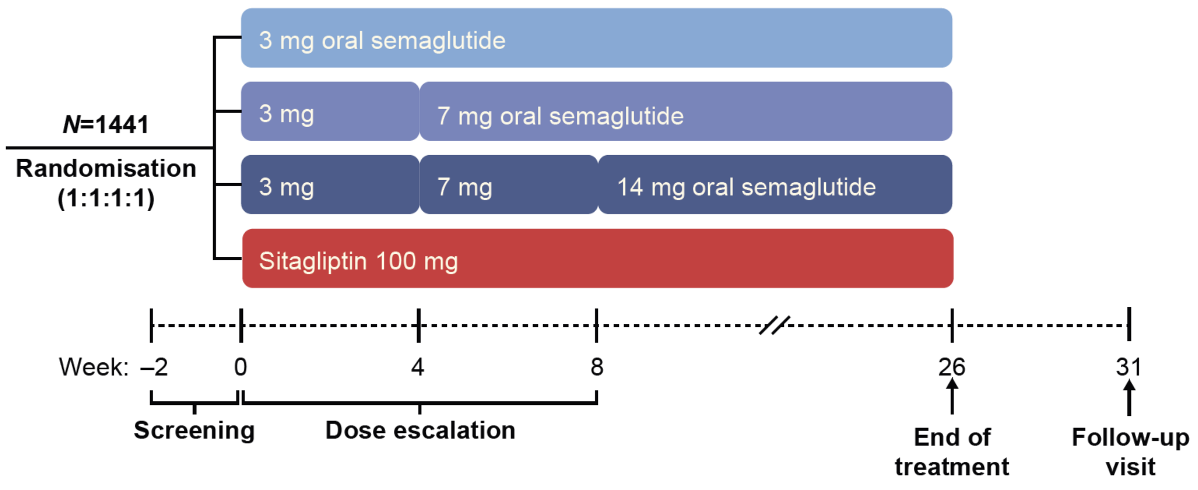

ESM Fig. 2 Hierarchical statistical testing strategy

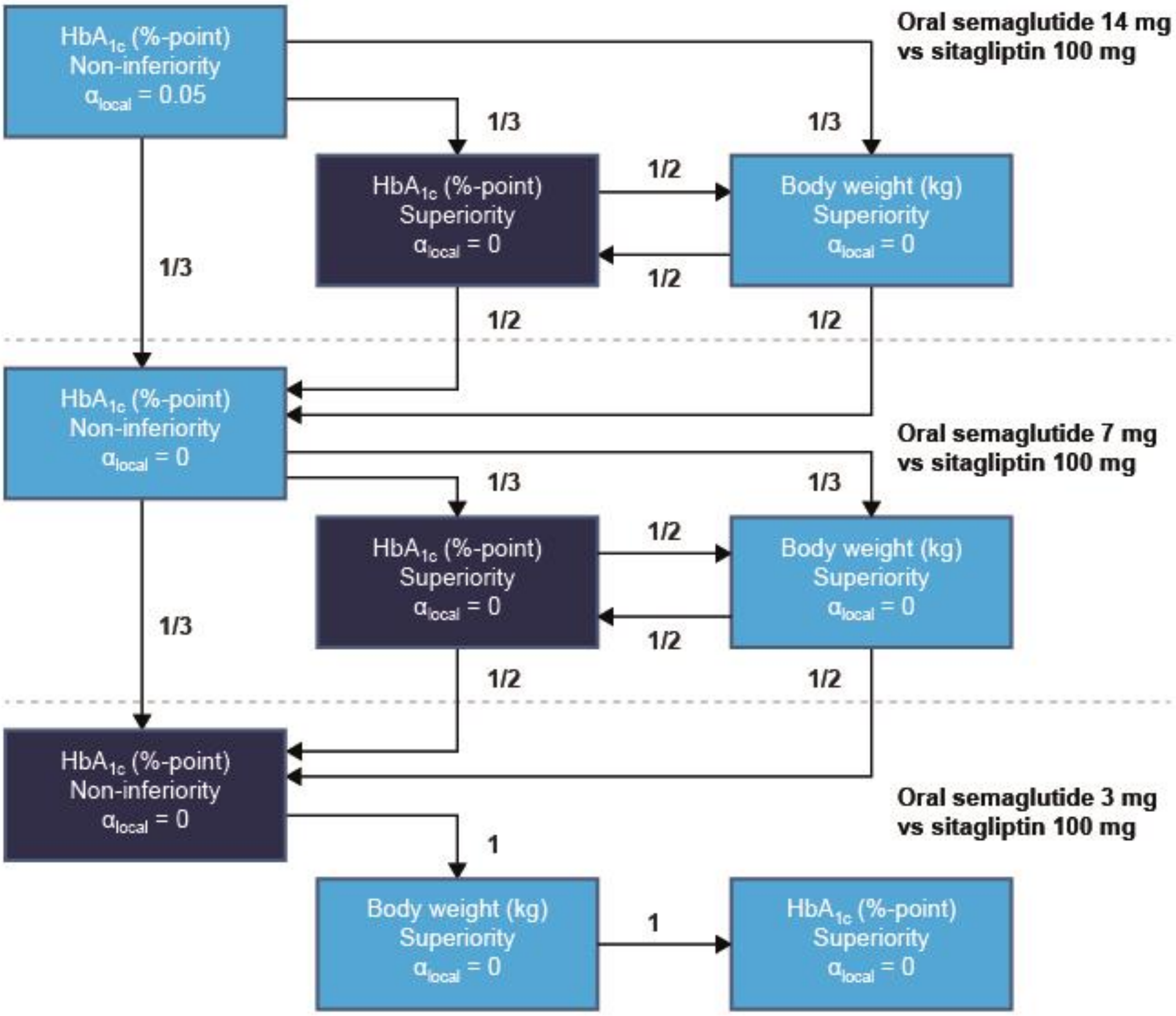


The overall significance level of α=0.05 (two-sided) was initially allocated to the $HbA_{1c}$ non-inferiority test of semaglutide 14 mg vs sitagliptin 100 mg. The local significance level (α-local) was to be reallocated if a hypothesis was confirmed according to the weight given by the directed edges between nodes (hypotheses). The sample size was based on the hypotheses in the dark boxes.

ESM Fig. 3 Change in fasting plasma glucose and 7-point self-monitored plasma glucose profile

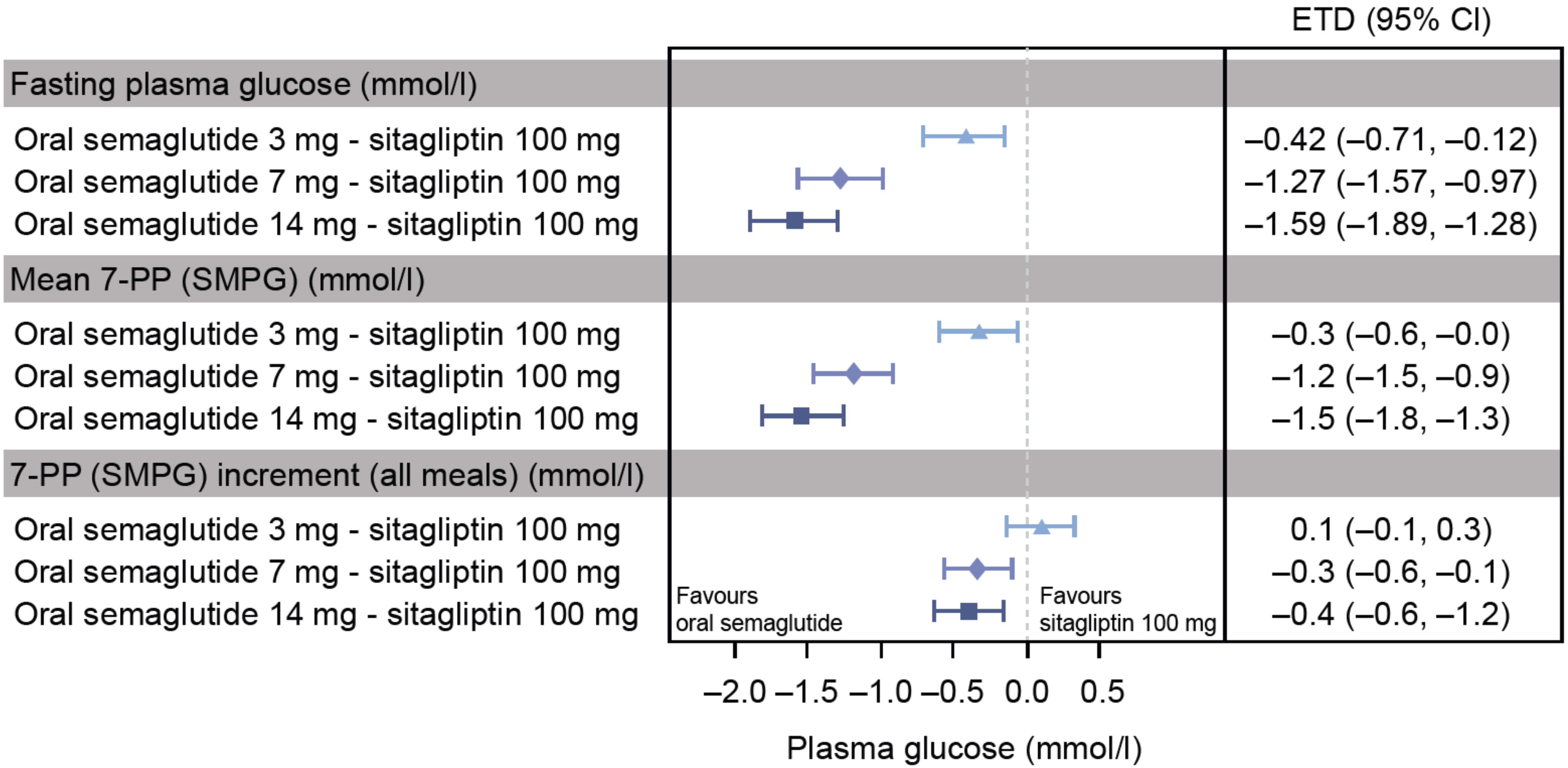


7-PP, 7-point profile; ETD, estimated treatment difference; SMPG, self-monitored plasma glucose
